# A multiscale model of the action of a capsid assembly modulator for the treatment of chronic hepatitis B

**DOI:** 10.1371/journal.pcbi.1012322

**Published:** 2025-05-06

**Authors:** Sarafa A. Iyaniwura, Tyler Cassidy, Ruy M. Ribeiro, Alan S. Perelson

**Affiliations:** 1 Theoretical Biology and Biophysics, Theoretical Division, Los Alamos National Laboratory, Los Alamos, New Mexico, United States of America; 2 School of Mathematics, University of Leeds, Leeds, United Kingdom; University of Tennessee Health Science Center College of Medicine Memphis, UNITED STATES OF AMERICA

## Abstract

Chronic hepatitis B virus (HBV) infection is strongly associated with increased risk of liver cancer and cirrhosis. While existing treatments effectively inhibit the HBV life cycle, viral rebound frequently occurs following treatment interruption. Consequently, functional cure rates of chronic HBV infection remain low and there is increased interest in a novel treatment modality, capsid assembly modulators (CAMs). Here, we develop a multiscale mathematical model of CAM treatment in chronic HBV infection. By fitting the model to participant data from a phase I trial of the first-generation CAM vebicorvir, we estimate the drug’s dose-dependent effectiveness and identify the physiological mechanisms that drive the observed biphasic decline in HBV DNA and RNA, and mechanistic differences between HBeAg-positive and negative infection. Finally, we demonstrate analytically and numerically that the relative change of HBV RNA more accurately reflects the antiviral effectiveness of a CAM than the relative change in HBV DNA.

## Author summary

Capsid assembly modulators (CAMs) are a novel class of anti-hepatitis B virus (HBV) treatments in clinical trials. These CAMs have a distinct mechanism of action from nucleos(t)ide analogues and thus represent an attractive option for the treatment of chronic HBV infection. We developed a multiscale model of the intracellular HBV lifecycle and extracellular dynamics using a time-since-infection structured partial differential equation. We fit the model to participant data from a recent phase I trial, performed a detailed analysis of the parameter estimates, identified key mechanisms driving viral response to first-generation CAM treatment, and demonstrated that HBV RNA dynamics impart more information regarding CAM effectiveness than HBV DNA dynamics, highlighting the potential role of HBV RNA as an informative indicator of CAM effectiveness.

## Introduction

Despite the availability of an effective vaccine, chronic hepatitis B virus infection (CHB) imposes a major burden on health systems worldwide and is estimated to contribute to one million deaths per year [1]. Often referred to as a silent epidemic [2], the World Health Organization estimated that over 250 million individuals worldwide were living with CHB in 2022 [3]. While effective antiviral therapies, such as pegylated interferon-α and nucleos(t)ide analogues (NAs) exist, interferon-α treatment is associated with an unfavourable toxicity profile and NA treatment has a low functional cure rate [4, 5]. NA treatment typically leads to complete suppression of circulating hepatitis B virus (HBV) DNA, however viral rebound frequently occurs upon treatment interruption [5, 6] which necessitates life-long treatment in many infected individuals. There has therefore been increased interest in the development of new HBV antivirals that could be used as monotherapy or, more likely, as part of combination therapies.

A novel class of HBV antivirals with a distinct mechanism of action from NAs, capsid assembly modulators (CAMs), have demonstrated promising results in recent clinical trials [4, 7–9]. CAMs interfere with a crucial step in the HBV viral life cycle by inhibiting the encapsidation of pregenomic RNA (pgRNA) [4, 9, 10]. By blocking the encapsidation of pgRNA and the resulting production of HBV DNA, CAM treatment has been shown to drive significant declines in HBV RNA and HBV DNA serum concentrations [4, 7, 9, 11]. Here, we consider a phase I trial of the first-generation CAM vebicorvir [4] and we analyze the antiviral efficacy of vebicorvir by developing a multiscale mathematical model of CAM treatment in the context of CHB.

Mathematical modeling has provided extensive insight into the viral dynamics of both hepatitis B and C as well as other viruses [12–19, 29]. The majority of existing models focus on extracellular quantities, such as HBV DNA or HCV RNA, which can be immediately compared against clinical data [12, 13, 73]. These models have provided valuable insight into the development of drug-resistance and treatment efficacy in hepatitis C infection [12, 23, 24]. Further, multiscale models, which characterize both the intracelluar and extracelluar viral dynamics, and thus permit a more precise representation of the mechanism of action of novel therapies, have been established to understand viral kinetics during treatment of hepatitis C [20, 21, 25–28]. However, much of the existing modeling in hepatitis B has focused on the dynamics of HBV DNA without explicitly considering the intracellular processes that comprise the HBV viral life cycle [22, 29–35]. This modeling has identified increased death rates of infected hepatocytes in HBe antigen (HBeAg) negative infections, highlighted the role of HBeAg status as a significant predictor of extracelluar viral dynamics, and has characterized the decay kinetics of HBV DNA during treatment. Nevertheless, recent experimental and modeling work has highlighted the role of HBV RNA as an important biomarker in understanding CHB treatment efficacy [36–38]. For example, Gonçalves et al. [39] developed a multiscale model of HBV infection that explicitly includes intracellular pgRNA and relaxed circular DNA (rcDNA) dynamics as well as circulating HBV DNA and RNA. They used the model to understand clinical data following treatment with the CAM, RG7907, or the NA, entecavir [39].

Here, we develop a multiscale model of HBV infection similar to the model developed by Gonçalves et al. [39]. Specifically, we explicitly consider the dynamics of intracelluar HBV encapsidated pgRNA and rcDNA and tie these dynamics to the extracellular dynamics of HBV RNA and HBV DNA. Unlike Gonçalves et al. [39], we incorporate the dynamics of uninfected hepatocytes and alanine aminotransferase (ALT). As shown by [40, 41], ALT dynamics can facilitate parameter identification in mathematical models of HCV infection and is commonly used as a biomarker of liver damage [42, 43]. We fit our multiscale model to the HBV RNA, HBV DNA, and ALT data from a multiple ascending dose monotherapy trial of vebicorvir [4]. We then use our model to identify the effect of vebicorvir treatment on HBV RNA and HBV DNA dynamics, identify mechanistic differences between HBeAg-positive and HBeAg-negative infections, identify the intracellular mechanisms that contribute to viral decline during treatment, and evaluate HBV RNA and HBV DNA as biomarkers of CAM effectiveness.

## Methods

### Viral load data

Our study uses longitudinal viral measurements made on days 0, 1, 7, 14, 21, 28, 35, 42, and 56 from the phase 1, randomized, placebo-controlled, multiple ascending dose study of the first-generation CAM, vebicorvir (Trial identifier: NCT02908191) [4]. Briefly, 32 participants with CHB and no anti-HBV therapy in the 3 months preceding the trial received either 100 mg (*n* = 10), 200 mg (*n* = 10), 300 mg (*n* = 10), or 400 mg (*n* = 2) oral doses of vebicorvir daily for 28 days and then were followed for another 28 days off therapy. The majority (*n* = 17) of participants were HBeAg-positive with further inclusion criteria reported by Yuen et al. [4].

One of the two participants in the 400 mg dose cohort discontinued treatment following an adverse event [4]. We therefore excluded the 400 mg dose cohort as only one participant completed the trial. In addition, we excluded an individual in the 300 mg dose cohort due to a pre-existing known CAM resistance mutation (Thr109Met) [4]. The remaining 29 participants in our study were in the 100 mg (*n* = 10), 200 mg (*n* = 10), and 300 mg (*n* = 9) dosing groups, with six, five, and six HBeAg-positive individuals in the 100 mg, 200 mg, and 300 mg dose cohorts, respectively.

The lower limit of detection (LLoD) for HBV DNA was 0.95 $\log_{10}$ IU/mL while the lower limit of quantitation (LLoQ) was 1.28 $\log_{10}$ IU/mL [4]. To convert from IU/mL to copies/mL, we used the standard conversion factor of 5.82 copies/IU HBV DNA [44]. We fit the model to HBV DNA and RNA data both expressed in units of copies/ml but present the HBV DNA data in their conventional units of IU/ml. Finally, Yuen et al. [4] used two independent assays for HBV RNA concentrations; we use the LLoD of 2.49 $\log_{10}$ copies/mL, which corresponds to the more sensitive of the two assays. Vebicorvir treatment decreased serum HBV RNA and HBV DNA concentrations. We calculated the minimal estimate for the mean decrease in HBV RNA and HBV DNA by replacing any measurements below the LLoD by the value of the LLoD. Across all dose levels, this minimal estimate for the mean decrease in HBV RNA and HBV DNA was 1.3 $\log_{10}$ copies/mL and 2.1 $\log_{10}$ IU/mL, respectively, during the 28 day treatment period. Rebound to approximately pre-treatment baseline serum HBV RNA and HBV DNA concentrations occurred rapidly following treatment cessation.

### Multiscale model of chronic HBV infection

Our multiscale model of CHB incorporates the major features of both the intracellular life cycle and extracellular dynamics of HBV. Broadly speaking, we model the intracellular dynamics of encapsidated pgRNA and rcDNA within infected hepatocytes, which allows us to accurately represent the mechanism of action of vebicorvir. Further, we model the extracellular dynamics of infected and uninfected hepatocytes, HBV RNA and DNA, and ALT.

**Fig 1:**
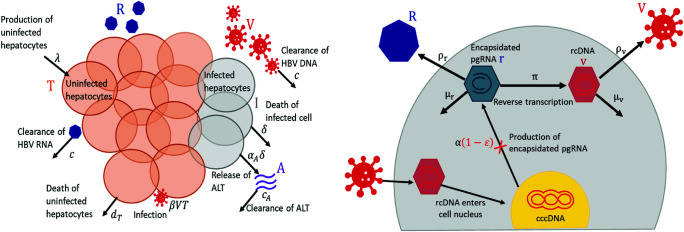
Schematic illustration of the multiscale model. Left panel: HBV extracellular dynamics, where uninfected hepatocytes (*T*) are produced at a constant rate λ and die at per capita rate *d*_*T*_. Hepatocytes become infected cells (*I*), following infection by HBV DNA (*V*). Infected cells are lost at per capita rate δ and secrete HBV RNA (*R*), HBV DNA, and ALT (*A*) at constant rates. The HBV RNA and HBV DNA are cleared at rates *c*_*r*_ and $c_{v}$, respectively. Right panel: HBV intracellular life cycle, which begins with a hepatocyte being infected and the release of rcDNA into the cell cytoplasm following the disintegration of its capsid. This rcDNA enters the cell nucleus and is converted to cccDNA. Encapsidated pgRNA (*r*) is produced by cccDNA at rate α. The encapsidated pgRNA is assembled into membrane bound particles and secreted as HBV RNA by the infected cell at rate $\rho_{r}$, decays at rate $\mu_{r}$, or is reverse transcribed into encapsidated rcDNA (*v*) at rate π. The rcDNA is either assembled into viral particles and secreted into the circulation at rate $\rho_{v}$ or decays at rate $\mu_{v}$ in the cell. Treatment with vebicorvir inhibits the production of encaptidated pgRNA with an effectiveness of ε (red cross in the right panel).

As has been shown previously in hepatitis C [40], modeling ALT dynamics can inform estimates of infected hepatocyte lifespans. Moreover, as hepatocytes are able to proliferate to counter liver damage, we do not expect the hepatocyte population to be constant. Therefore, we explicitly include the dynamics of the uninfected hepatocytes in our model. A schematic representation of our model is given in Fig 1.

At the extracellular scale, uninfected hepatocytes (*T*) are produced at a constant rate λ and cleared linearly with per capita rate *d*_*T*_. Hepatocytes are infected by HBV DNA containing particles (*V*) with rate constant β. These infected hepatocytes die with per capita death rate δ and produce HBV RNA containing particles (*R*) and HBV DNA containing virions, which are cleared at per capita rates *c*_*r*_ and $c_{v}$, respectively. In principle, death of infected hepatocytes could be driven by cytolytic T cells (CTLs), which we do not include in our model. CTL involvement typically results in an “ALT flare,” where the ALT concentration increases by orders of magnitude. None of the participants in this study exhibited an ALT flare and thus we assume that if CTLs have any effect on infected cells, this effect is approximately constant for the duration of the trial and included in the constant parameter δ.

As mentioned, we explicitly model the intracellular processes leading to HBV RNA and HBV DNA production. We keep track of the time since infection (or infection age) of HBV infected hepatocytes using an age-structured partial differential equation (PDE), where the density of infected cells with infection age *a* at time *t* is given by *i*(*t*,*a*). Following infection, HBV rcDNA is converted to cccDNA in the nucleus of infected hepatocytes [6]. This cccDNA forms a stable template for the production of encapsidated HBV pgRNA. We assume the production of encapsidated pgRNA from cccDNA occurs at a constant rate α and denote the amount of intracellular encapsidated pgRNA in an infected hepatocyte with infection age *a* by *r*(*t*,*a*). Encapsidated pgRNA decays intracellularly at rate $\mu_{r}$, is reverse transcribed into encapsidated rcDNA, *v*(*t*,*a*), with rate constant π, or is secreted by infected cells as enveloped HBV RNA containing particles into the circulation at rate $\rho_{r}$ per cell. The rate at which encapsidated pgRNA enters the circulation as HBV RNA is $\rho_{r}P(t)$, where *P*(*t*) is the total amount of pgRNA in infected cells and is given by


$$P(t)=\int_{0}^{\infty }r(t,a)\;i(t,a)\;da.$$ (1)

Following reverse transcription of intracellular encapsidated pgRNA, intracellular rcDNA either decays at rate $\mu_{v}$ or is assembled into viral particles containing HBV DNA and secreted with rate $\rho_{v}$ per cell. The total rate at which HBV DNA containing particles are released into the circulation is given by $\rho_{v}C(t)$, where *C*(*t*) represents the total amount of encapsidated rcDNA in infected cells, given by


$$C(t)=\int_{0}^{\infty }v(t,a)\;i(t,a)\;da.$$ (2)

Finally, we explicitly model the dynamics of ALT, where *A*(*t*) represents its serum concentration. Following Cardozo et al. [40], we assume that ALT is produced at a constant background rate *s*, independently of the death of infected hepatocytes, is cleared with rate *c*_*A*_, and is released at a constant rate $\alpha_{A}\delta$ due to the death of infected cells. Note $\alpha_{A}$ is the amount of ALT released when an infected cell dies.

Taken together, the equations describing the multiscale model are


$$\begin{array}{cc} \frac{\text{d}}{\text{dt}}T(t) & =\lambda -d_{T}T(t)-\beta T(t)V(t),\\ \left(\partial_{t}+\partial_{a})i(t,a\right) & =-\delta i(t,a),\\ \left(\partial_{t}+\partial_{a})r(t,a\right) & =\alpha -\left(\mu_{r}+\rho_{r}+\pi \right)r(t,a),\\ \left(\partial_{t}+\partial_{a})v(t,a\right) & =\pi r(t,a)-\left(\mu_{v}+\rho_{v}\right)v(t,a),\\ \frac{\text{d}}{\text{dt}}R(t) & =\rho_{r}\int_{0}^{\infty }r(t,a)i(t,a)\;\text{d}a-c_{r}R(t),\\ \frac{\text{d}}{\text{dt}}V(t) & =\rho_{v}\int_{0}^{\infty }v(t,a)i(t,a)\;\text{d}a-c_{v}V(t),\\ \frac{\text{d}}{\text{dt}}A(t) & =s+\alpha_{A}\delta \int_{0}^{\infty }i(t,a)\;\text{d}a-c_{A}A(t). \end{array}\;\}$$ (3)

Newly infected cells have infection age *a* = 0, and the density of newly infected cells at time *t* is given by i(t,0)=βV(t)T(t). We assume that newly infected cells have no intracellular pgRNA or rcDNA, i.e., r(t,0)=0 and v(t,0)=0, as the rcDNA from the initial virion that successfully infected an hepatocyte must have been transported to the nucleus and converted to cccDNA, and thus behaves differently than newly produced rcDNA that can be converted into virions or degraded.

In principle, the rate constants describing intracellular processes within an infected cell, as well as the death rate of infected cells, could depend on the infection age of the cells. For example, Nelson et al. [45], examined an HIV model in which the rate of viral production and the death rate of productively infected cells varies with their infection age. Similarly, Hailegiorgis et al. [46] developed an agent-based model of acute HBV infection in which the rate of virion production increased until reaching a constant rate. As little information is available on the age-dependence of the parameters in our model, we restrict our analysis to the case of age-independent parameters. In this case, the multiscale PDE model 3) can be transformed into the following ordinary differential equation (ODE) system by integrating over the age structure as previously shown [18, 19, 39, 47]


$$\begin{array}{cc} \frac{\text{d}T}{\text{d}t} & =\lambda -\beta VT-d_{T}T,\\ \frac{\text{d}I}{\text{d}t} & =\beta VT-\delta I,\\ \frac{\text{d}P}{\text{d}t} & =\alpha I-(\mu_{r}+\delta +\pi +\rho_{r})P,\\ \frac{\text{d}C}{\text{d}t} & =\pi P-(\mu_{v}+\delta +\rho_{v})C,\\ \frac{\text{d}R}{\text{d}t} & =\rho_{r}P-c_{r}R,\\ \frac{\text{d}V}{\text{d}t} & =\rho_{v}C-c_{v}V,\\ \frac{\text{d}A}{\text{d}t} & =s+\alpha_{A}\delta I-c_{A}A. \end{array}\}$$ (4)

Here, *I*(*t*) is the total concentration of infected hepatocytes defined by


$$I(t)=\int_{0}^{\infty }i(t,a)\;da,$$

while *P*(*t*) and *C*(*t*) are given in Eqs (1) and (2), respectively. The ODE system Eq (4) is a mathematically equivalent and numerically tractable representation of the PDE model, Eq (3), under the assumption of age-independent parameters. We refer to the ODE system Eq (4) as the *pre-treatment/baseline model* throughout this study.

#### Modeling vebicorvir effectiveness

In the phase I trial of vebicorvir [4], participants received vebicorvir once daily for 28 days and then were followed for another 28 days. We neglect drug pharmacokinetics during the daily dosing period as the drug was rapidly absorbed [4]. We assume that vebicorvir inhibits pgRNA encapsidation immediately following dosing and leads to the production of empty capsids, consistent with its classification as a type-E (empty capsid) CAM. Thus, during the treatment period, we model the effect of vebicorvir as reducing the production rate of encapsidated pgRNA, α, by a constant factor $\left(1-\varepsilon_{c}\right)$, where $\varepsilon_{c}\in [0,1]$ represents the CAM effectiveness and its value depends on the dose of the CAM.

During treatment we assume the concentration of vebicorvir is at a dose-dependent steady-state concentration ${\text{C}}^{*}$. At the end of treatment, we assume that the drug washes out and its concentration decays exponentially at rate *k*. Then, during drug washout, we use a maximum effect, or Emax model, for vebicorvir’s effectiveness given by


$$\varepsilon (t)=\frac{C^{*}e^{-k(t-\tau )}}{C^{*}e^{-k(t-\tau )}+EC_{50}},\;t>\tau ,$$ (5)

where τ=28 days is the duration of the treatment period and *EC*_50_ is the concentration of vebicorvir that gives half of the maximum effectiveness of the drug. To calculate the ratio of the *EC*_50_ to ${\text{C}}^{*}$ at a given dose level, we note that $\varepsilon (\tau )=\varepsilon_{c}$, where $\varepsilon_{c}$ is the drug effectiveness during therapy. We can then re-arrange Eq (5) to find


$$\frac{C^{*}}{EC_{50}}=\frac{\varepsilon_{c}}{1-\varepsilon_{c}},$$

and using this identity gives


$$\varepsilon (t)=\frac{C^{*}e^{-k(t-\tau )}}{C^{*}e^{-k(t-\tau )}+EC_{50}}=\frac{\varepsilon_{c}\;e^{-k(t-\tau )}}{\varepsilon_{c}\;\left(e^{-k(t-\tau )}-1\right)+1}.$$

Incorporating the waning vebicorvir effects during drug wash-out only necessitates estimating the clearance rate, *k*. We also considered an approach where the drug effectiveness, ε(t), is set to zero immediately after treatment cessation (see S1 Text).

Incorporating vebicorvir mediated inhibition of pgRNA encapsidation into the baseline model Eq (4) gives


$$\frac{\text{d}T}{\text{d}t}=\lambda -\beta VT-d_{T}T,$$ (6)

$$\frac{\text{d}I}{\text{d}t}=\beta VT-\delta I,$$ (7)

$$\frac{\text{d}P}{\text{d}t}=\alpha (1-\varepsilon (t))I-(\mu_{r}+\delta +\pi +\rho_{r})P,$$ (8)

$$\frac{\text{d}C}{\text{d}t}=\pi P-(\mu_{v}+\delta +\rho_{v})C,$$ (9)

$$\frac{\text{d}R}{\text{d}t}=\rho_{r}P-c_{r}R,$$ (10)

$$\frac{\text{d}V}{\text{d}t}=\rho_{v}C-c_{v}V,$$ (11)

$$\frac{\text{d}A}{\text{d}t}=s+\alpha_{A}\delta I-c_{A}A,$$ (12)

where the drug effectiveness over the entire trial, ε(t), is defined by


$$\varepsilon (t)=\{\begin{array}{cc} \varepsilon_{c} & \;t⩽\tau ;\\ \frac{\varepsilon_{c}e^{-k(t-\tau )}}{\varepsilon_{c}(e^{-k(t-\tau )}-1)+1} & \;t>\tau . \end{array}$$ (13)

We refer to the ODE model, Eqs (6)–(12), as the *treatment model*.

#### Initial conditions corresponding to chronic HBV infection

Since we are interested in chronic HBV infection, we assume that the viral dynamics model is in steady-state prior to treatment. We thus use the steady-state solutions of the baseline model Eq (4) as the initial conditions of the treatment model Eqs (6)–(12). The baseline viral load, $V_{0}$, is given by


$$V_{0}=\frac{\lambda \;\rho_{v}\;ℳ}{c_{v}}-\frac{d_{T}}{\beta },$$

where $ℳ=\pi \;\alpha /(\delta \;\psi_{1}\;\psi_{2})$, with $\psi_{1}=\mu_{r}+\delta +\pi +\rho_{r}$ and $\psi_{2}=\mu_{v}+\delta +\rho_{v}$. The remaining steady-state concentrations can be written in terms of $V_{0}$ as follows


$$T_{0}=\frac{\lambda }{\beta V_{0}+d_{T}},\;I_{0}=\frac{\beta V_{0}T_{0}}{\delta },\;P_{0}=\frac{\alpha I_{0}}{\psi_{1}},$$

$$C_{0}=\frac{c_{v}}{\rho_{v}}V_{0},\;R_{0}=\frac{\rho_{r}P_{0}}{c_{r}},\;\text{and}\;A_{0}=\frac{1}{c_{A}}\left(s+\frac{\alpha_{A}\;c_{v}}{\rho_{v}\;ℳ}V_{0}\right).$$

We calculate these expressions explicitly in terms of the model parameters in S1 Text. We consider *t* = 0 as the beginning of CAM treatment and set


$$T(0)=T_{0},\;I(0)=I_{0},\;P(0)=P_{0},\;C(0)=C_{0},\;R(0)=R_{0},\;\text{and}\;V(0)=V_{0}.$$

Finally, we note that imposing that the viral dynamics model is in steady-state prior to treatment yields natural candidates for initial densities $i_{0}(a),r_{0}(a),$ and $v_{0}(a)$ of the age-structured PDE model. Specifically, the initial density of infected hepatocytes at time *t* = 0 represent precisely those hepatocytes that were infected at *t*<0 and have not been cleared in the intervening time. Following Cassidy et al. [48], it is possible to map the initial densities backwards along the characteristic line, and using the assumption that pre-treatment the system is in steady state, obtain explicit expressions for $i_{0}(a),r_{0}(a),$ and $v_{0}(a)$ as functions of the baseline viral load and uninfected hepatocyte concentration.

### Statistical and error model for parameter estimation

We estimate model parameters using a non-linear mixed effects modeling framework implemented in Monolix software version 2021R1 [49]. Below, we give the details of the statistical and error models used in our fitting.

#### Statistical model

We assume that the majority of our structural model parameters $\phi_{i}$ are log-normally distributed. The individual parameters that follow a log-normal distribution are defined as


$$\phi_{i}=\varphi \;e^{\left(\psi_{i}+\eta_{i}\right)},$$ (14)

where φ is the population estimate, $\psi_{i}\sim 𝒩(0,\omega^{2})$ represents the random effects corresponding to inter-individual variability, and $\eta_{i}$ is a potential covariate vector for individual i [49].

The parameter capturing vebicorvir effectiveness, ε, has natural upper and lower bounds, namely ε∈[0,1]. Thus, we assume that ε follows a logit-normal distribution in the open interval (0,1). Then, the individual estimates for ε are defined as


$$\phi_{i}=\;\frac{\varphi e^{\left(\psi_{i}+\eta_{i}\right)}}{1+\varphi \;\left(e^{\left(\psi_{i}+\eta_{i}\right)}-1\right)},$$ (15)

where φ, $\eta_{i}$ and $\psi_{i}$ are as defined in Eq (14).

We tested multiple covariate structures for the population estimates of different parameters, including effects of the categorical variables “HBeAg status” and “vebicorvir dose” in $\eta_{i}$. Details and results are described in the S1 Text. We also included a correlation between α and β, as suggested by Monolix.

#### Error model

We simultaneously fit Eqs (10), (11), and (12) to the HBV RNA, HBV DNA, and ALT data, respectively. These biomarkers are sampled at *j* = 9 distinct time points *t*_*j*_ in the phase I trial of vebicrovir. For each participant, we consider *k* = 3 observations, corresponding to the HBV DNA, HBV RNA, and ALT data, respectively. Accordingly, for the *i*–th participant, we denote this data by *Y*_*i*,*j*,*k*_ and the vector of model parameters by $\phi_{i}$.

Then, for each participant, we define the statistical model


$$Y_{i,j,k}=f(t_{i,j,k},\phi_{i})+g(f(t_{i,j,k},\phi_{i}),\gamma )\;e_{i,j,k},$$

where $f(t_{i,j,k},\phi_{i})$ represents the structural model output for participant *i*, given by Eqs 6 to 12. In this framework, the error model is given by $g(f(t_{i,j,k},\phi_{i}),\gamma )e_{i,j,k}$, where *e*_*i*,*j*,*k*_ is the residual error of participant *i* at measurement *j* for biomarker *k*, and γ is a vector of parameters which depends on the specific error model. Here, *e*_*i*,*j*,*k*_ follows a normal distribution with mean 0 and variance 1. The error function $g(f(t_{i,j,k},\phi_{i}),\gamma )$ determines the variance of the residual error model [50]. We consider ALT, HBV RNA, and HBV DNA concentrations on the logarithmic scale. For HBV RNA and HBV DNA, we set


$$\log Y_{i,j,k}=\log f(t_{i,j,k},\phi_{i})+a_{k}e_{i,j,k}\;\text{for}\;k=1,2,$$

which linearly scales the residual error *e*_*i*,*j*,*k*_ by the error parameter *a*_*k*_, which is estimated during our parameter fitting. As viral load is measured in terms of the number of PCR amplification cycles, the viral load measurements have a proportional error, that is multiplicative, on the linear scale. Consequently, this error is additive on the logarithmic scale as is common in many models of viral dynamics [39, 51–54]. We fit the ALT data using a proportional error model


$$\log Y_{i,j,3}=\log f(t_{i,j,3},\phi_{i})+a_{3}\;\log f(t_{i,j,3},\phi_{i})\;e_{i,j,3},$$

where *a*_3_ is an unknown error parameter to be estimated.

### Model fitting and parameter estimation

We solve the multiscale model (Eqs (6) to (12)) numerically, and fit the model solutions simultaneously to the longitudinal HBV RNA (*R*(*t*)), HBV DNA (*V*(*t*)), and ALT (*A*(*t*)) measurements of the 29 participants in our study. The mixed-effect approach uses a total of 783 data points, from all 29 participants, to fit our model and estimate the model parameters for each individual participant. Consequently, the model dynamics are informed by the entirety of the participant data, even for participants, such as ID:26, who do not have any HBV RNA measurements above the LLoD.

Parameter estimation was performed by maximizing the likelihood estimator using the stochastic approximation expectation-maximization (SAEM) algorithm [57] implemented in Monolix [49]. The log-likelihood was calculated using the importance sampling Monte Carlo method. HBV RNA measurements below the LLoD of 2.49 $\log_{10}$ copies/mL and HBV DNA measurements below the LLoD of 0.95 $\log_{10}$ IU/mL were left-censored.

#### Fixed parameters

We fixed some of the model parameters to estimates from the literature to reduce the number of free parameters in our model. Based on the estimate that, in the absence of infection, the liver has $2\times 10^{11}$ hepatocytes [58], we assumed the hepatocyte concentration is $T_{ue}\approx 1.3\times 10^{7}$ cells/mL, as was previously done [12]. Uninfected hepatocytes have a roughly 6 month (~180 days) half-life [59], which corresponds to a per capita death rate of $d_{T}=\log_{e}(2)/180\approx 0.004\;\text{/day}$. In the absence of infection, the steady-state concentration of hepatocytes is given by $T_{ue}=\lambda /d_{T}$ so $\lambda =d_{T}\;T_{ue}=5.2\times 10^{4}\;\text{cells/mL/day}$.

#### Estimated parameters

At the extracellular scale, we estimated the infection rate (β) and the death rate of infected cells (δ). We also estimated the intracellular production rate of encapsidated pgRNA (α), the reverse transcription rate of encapsidated pgRNA to rcDNA (π), and the secretion rates of HBV RNA ($\rho_{r}$) and HBV DNA ($\rho_{v}$). Additionally, we estimated both the effectiveness, ε, and clearance rate, *k*, of vebicorvir.

In the ALT ODE, Eq (12), there are three unknown ALT specific parameters that need to be estimated, the ALT background production rate, *s*, the ALT clearance rate, *c*_*A*_, and the ODE initial condition, i.e., the pretreatment level of ALT, *A*_0_. As we have no a priori estimate of *s*, we chose to estimate the baseline level of ALT in the absence of infection (*A*_*ue*_), which is typically below the upper limit of normal of approximately 40 IU/L. In the absence of infection, the ODE for ALT reduces to


$$\frac{dA}{dt}=s-c_{A}A.$$

At the uninfected steady state, $s=c_{A}\;A_{ue}$ and thus *s* can be computed from estimates of *c*_*A*_ and *A*_*ue*_. This estimate for *s* corresponds to ALT production that results from natural turnover of hepatocytes as well as production of low levels of ALT by cells other than hepatocytes, such as muscle cells [72], and may differ between individuals. At the pre-treatment steady-state *A*_0_, the amount of ALT released when an infected cell dies is given by (see Eq (12))


$$\alpha_{A}=\frac{c_{A}A_{0}-s}{\delta I_{0}},$$

where *I*_0_ is the baseline number of infected hepatocytes at treatment initiation and *A*_0_ is the ALT concentration at treatment initiation, which we estimate from the viral load data.

#### Parameter identifiability analysis

We used the baseline concentrations of HBV DNA, HBV RNA, and the ratio between these measurements to demonstrate how the available viral load data identify the model parameters. Further, we also estimate the rate of viral rebound following treatment cessation using a quasi-steady state assumption. Finally, we adapted the likelihood continuation technique from [63] to quantify the dependence of each estimated parameter on the viral load data. Further details are presented in S1 Text.

### Statistics

We used the Wilcoxon test [55] in R version 3.6.3 [56] to compare the distributions of the pre-treatment steady-states of the model variables for the HBeAg-positive and HBeAg-negative individuals.

## Results

### Model development

To test our model, given in Methods, against other alternatives, we tried different assumptions based on preliminary fitting of the models to the full data set (HBV DNA, HBV RNA and ALT). Initial results indicated that the export rates of particles containing encapsidated RNA and encapsidated DNA were very similar, which makes biological sense, since the physical properties of these particles are similar. Indeed, when we tested a model with $\rho_{v}=\rho_{r}=\rho$, we found that this model had a lower corrected Bayesian information criterion (BICc) value of 21.0 than when estimating these export rates separately, where the BICc was 28.9. Moreover, the estimate of the random effects for ρ was small, and a model with no random effects for this parameter had an even lower BICc (=12.1) [61, 62].

Biologically, we expect that the clearance rates of HBV RNA particles ($c_{v}$) and of HBV DNA particles (*c*_*r*_) are similar as these particles differ only in their internal content [36]. We tested this, by fitting preliminary models estimating $c_{v}$ and *c*_*r*_ separately, with and without random effects, and comparing the results with estimating only $c=c_{v}=c_{r}$. We found that the latter model was more parsimonious, with lower BICc (see Table D in S1 Text). Therefore, we assumed $c_{v}=c_{r}=c$ in the modeling that followed (as was also used before [39]). Still, estimating the clearance rate *c* is challenging from our data, so we followed Gonçalves et al. [39] and tested seven different fixed values of c=1,2,3,5,10,15, and 20/day based on previous studies [32, 60]. We found that *c* = 1/day provided the lowest BICc (see Table B in S1 Text).

Not having data to distinguish the intracellular degradation rates of encapsidated pgRNA and rcDNA, and as in [39], we assumed that these rates are identical, $\mu_{v}=\mu_{r}=\mu$. This intracellular degradation rate is difficult to estimate from our data which is taken from the circulation. We thus tested 13 fixed values of μ=0,0.05,0.1,0.2,...,1,2/day and found that the best-fit occurred with μ=0 (see Table B in S1 Text). Thus, based on this analysis, as well as for consistency with previous modeling [39] and the expectation that encapsidation protects molecules from degradation, we fixed μ=0.

We next tested the inclusion of random effects and covariates for different model parameters. We tested if the drug effectiveness, ε, depended on the vebicorvir dose. We also tested if various parameters differed between HBeAg-positive and HBeAg-negative infection. Finally, we also tested for correlations between estimated the parameters. A summary of these results can be found in Table C of S1 Text.

Finally, we tested alternative models, described in S1 Text, for the biology of HBV infection and the mechanism of action of vebicorvir. We found that none of the tested models improved the goodness of fit. More importantly, the population parameter estimates were very similar for models that provided the best fits, as assessed by BICc (see Table D, Fig F, and the corresponding discussion in S1 Text).

Overall, the model that we used going forward to analyze our data is based on Eqs (6) to (12), with $\rho_{v}=\rho_{r}=\rho$, $c_{v}=c_{r}=c=1$/day, $\mu_{v}=\mu_{r}=\mu =0$, and no random effects on ρ, *c*_*A*_, nor on any of the fixed parameters. Moreover, the model includes dose as a covariate on the drug effectiveness, ε, and HBeAg status as a covariate on the infection rate (β), the production rate of encapsidated pgRNA (α), and the death rate of infected cells (δ). This best model also included a negative correlation between the pgRNA production rate (α) and the virus infection rate (β) with coefficient –0.945.

The model parameters, units, and biological descriptions are summarized in Table 1.

**Table 1:** Model parameters and descriptions.

Parameter (unit)	Description	Value	Random effects
λ (cells/mL/day)	Production rate of hepatocytes	$5.2\times 10^{4}$	Fixed^1^
*d*_*T*_ (/day)	Death rate of uninfected hepatocytes	0.004	Fixed^1^
β (mL/copies/day)	Infection rate constant	Fitted	Yes
$\varepsilon_{c}$	Drug effectiveness during treatment	Fitted	Yes
δ (/day)	Death rate of infected cells	Fitted	Yes
α (copies/cell/ml/day)	Production rate of encapsidated pgRNA	Fitted	Yes
π (/day)	Reverse transcription rate of pgRNA to rcDNA	Fitted	Yes
ρ (/day)	Secretion rate of encapsidated pgRNA and rcDNA	Fitted	No
μ (/day)	Intracellular decay rate of encapsidated pgRNA and rcDNA	0	Varied^2^
*c* (/day)	Extracellular clearance rate of HBV RNA and HBV DNA	1	Varied^2^
*s* (U/L/day)	Baseline production rate of ALT	Fitted	Yes
$\alpha_{A}$ (U/L/mL/cell)	Amount of ALT released upon infected cell death	Calculated	No
*c*_*A*_ (/day)	Clearance rate of ALT	Fitted	No
*k* (/day)	Drug clearance rate	Fitted	Yes
τ (days)	Treatment duration	28	Fixed^1^

^1^ Fixed indicates that the population parameter was fixed prior to fitting. These parameters did not have random effects ^2^ Varied indicates that we tested different values for the indicated parameter before fixing the population estimate at the value indicated. These parameters did not have random effects. Further details are given in S1 Text.

### Model fits to participant data

**Fig 2:**
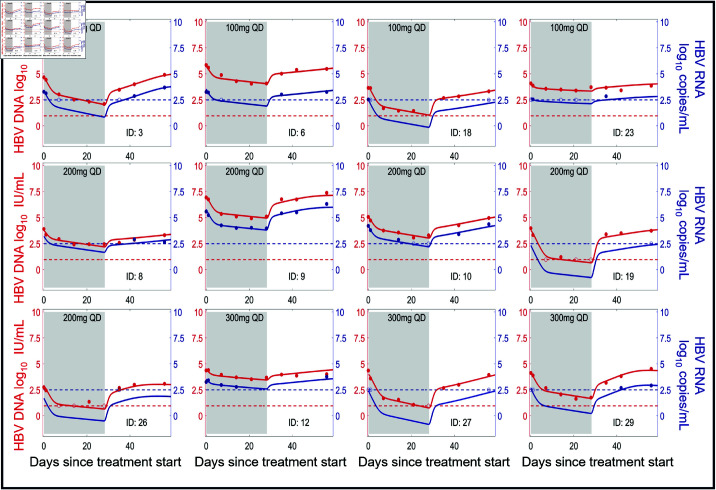
Model fits of HBV RNA and HBV DNA (HBeAg-negative group). Individual fits to the longitudinal HBV RNA (blue) and HBV DNA (red) measurements during treatment (shaded area) and follow-up using the multiscale model Eqs (6)–(12) with individual parameter estimates in Table A of S1 text. Dots are viral measurements, and solid lines are model predictions. Horizontal dashed lines represent the lower limit of detection (LLoD) of 0.95 $\log_{10}$ IU/mL for HBV DNA (red) and 2.49 $\log_{10}$ copies/mL for HBV RNA (blue). Open circles are viral measurements below the LLoD. The corresponding ALT fits for these participants are given in S1 Text Fig J. Participants 19, 26, and 27 have no HBV RNA measurements above the LLoD while participants 8 and 29 do not have HBV RNA measurements during treatment.

**Fig 3:**
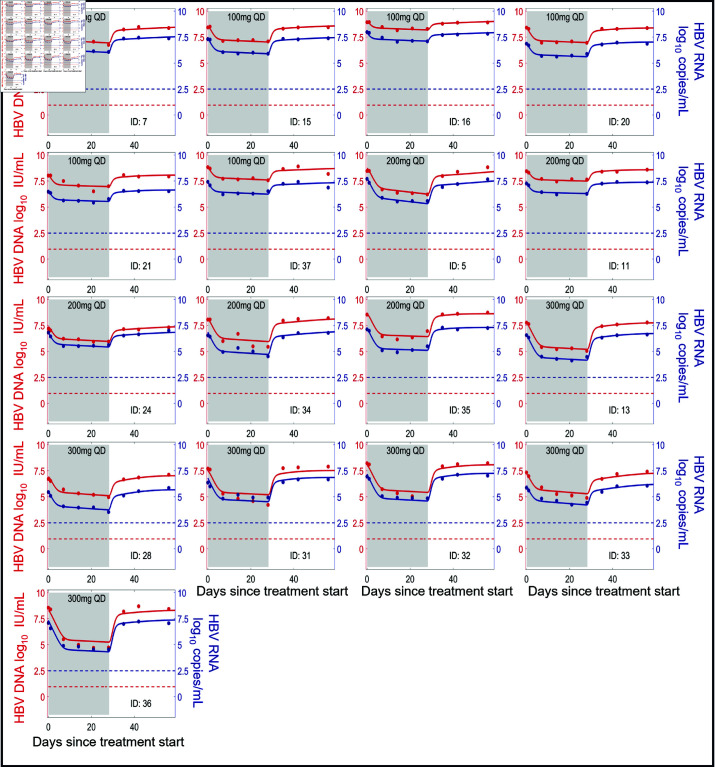
Model fits of HBV RNA and HBV DNA (HBeAg-positive group). Individual fits to the longitudinal HBV RNA (blue) and HBV DNA (red) measurements during treatment (shaded area) and follow-up using the multiscale model Eqs (6)–(12) with individual parameter estimates in Table A of S1 text. Dots are viral measurements, and solid lines are model predictions. Horizontal dashed lines represent the lower limit of detection (LLoD) of 0.95 $\log_{10}$ IU/mL for HBV DNA (red) and 2.49 $\log_{10}$ copies/mL for HBV RNA (blue). Open circles are viral measurements below the LLoD. The corresponding ALT fits for these participants are given in Fig K of S1 Text.

We show the individual fits of our model to the HBV RNA and HBV DNA data in Figs 2 and 3. The corresponding individual fits to ALT are shown in Figs J and K of S1 Text. In general, our model captures the viral dynamics in all participants both during treatment and during the viral rebound that follows treatment cessation.

The population-level parameters were well-estimated, as measured by the relative standard error, and are reported in Table 2. We identified a dose-dependent vebicorvir effect, with estimated efficacies of 90.7%, 97.0%, and 98.4% for the 100, 200, and 300 mg daily dose.

**Table 2:** Estimated population parameters. Fixed parameters do not have random effects and this is indicated as NA (Not Applicable), some parameters were estimated at the population level (without random effects), and this is indicated as –. R.S.E. is the relative standard error of the estimate. P-values were computed using the Wald test in Monolix and used to compare the population estimates for covariates.

Parameter	Fixed Effects (R.S.E., %)	Random Effects (R.S.E., %)
$\varepsilon_{c}$ (100 mg)	0.907 (4.55)	1.36 (14.3)
$\varepsilon_{c}$ (200 mg)^*1^	0.970 (1.52)	1.36 (14.3)
$\varepsilon_{c}$ (300 mg)^*2^	0.984 (0.90)	1.36 (14.3)
β (HBeAg-negative)^**^	$4.2\times 10^{-7}$ mL/copies/day (4.53)	0.92 (15.2)
β (HBeAg-positive)	$1.1\times 10^{-10}$ mL/copies/day (2.89)	0.92 (15.2)
α (HBeAg-negative)^***^	0.199 copies/cell/day (34.3)	0.81 (15.7)
α (HBeAg-positive)	390.8 copies/cell/day (9.23)	0.81 (15.7)
π	204.6/day (5.57)	0.35 (15.8)
δ (HBeAg-negative)${\;}^{†}$	0.070/day (14.4)	0.38 (34.1)
δ (HBeAg-positive)	0.025/day (28.9)	0.38 (34.1)
ρ	2.48/day (26.6)	–
*c*	1/day (fixed)	NA
μ	0/day (fixed)	NA
*c*_*A*_	0.057/day (26.4)	–
*A*_0_	38.4 U/L (3.59)	0.30 (13.6)
*A*_*ue*_	22.8 U/L (5.90)	0.27 (20.1)
*k*	2.29/day (230)	0.24 (71.6)

^*1^*p* = 0.059, ^*2^*p* = 0.006, ${\;}^{**}p=2.2\times 10^{-16}$, ${\;}^{***}p<2.2\times 10^{-16}$, ${\;}^{†}p=1.0\times 10^{-3}$.

Our identifiability analysis, given in S1 Text, demonstrates that the observable viral kinetics during treatment and following treatment cessation inform the unknown model parameters. We next tested if small perturbations in the observed data will influence individual parameter estimates using the likelihood continuation method [63]. We found that the estimates of the export rate ρ are sensitive to 10% changes in each of the HBV RNA and HBV DNA measurements. This parameter is particularly sensitive to viral load measurements taken during the final two weeks of treatment (Fig I of S1 Text). The likelihood continuation analysis also indicates that ρ and ε are informed by measurements during treatment [63]. Lastly, the initial conditions that correspond to the subjects being at steady-state pre-treatment are directly informed by the baseline viral load and strongly depend on β. In S1 Text, we demonstrate that these initial conditions, combined with the viral dynamics during treatment and following treatment cessation, are sufficient to link the model parameters with the observable viral dynamics. Taken together, our analysis indicates that our parameter estimates for the intracellular dynamics are well-informed by the available extracellular data.

We found no significant difference in the baseline ALT levels between the HBeAg-positive and HBeAg-negative groups, even though two HBeAg-positive individuals (ID: 5, 24) had elevated ALT at the start of treatment. The elevated baseline ALT levels in these individuals may indicate an anti-HBV immune response prior to treatment initiation. Their ALT levels declined during treatment and approached a similar level to the other participants by day 56 post-treatment initiation (Fig K of S1 Text). There were no significant changes in the ALT levels of the remaining participants throughout the study period.

### Mechanistic differences between HBeAg-positive and negative infection

HBeAg status is an important predictor of clinical progression with faster progression to liver disease observed in HBeAg-negative patients, despite persistently higher viral load in HBeAg-positive patients [32, 64]. We leveraged our multiscale model to identify the mechanistic differences between HBeAg-positive and HBeAg-negative participants. As expected, the baseline HBV DNA concentration, $V_{0}$, is significantly higher in HBeAg-positive participants ($1.3\times 10^{8}$ vs $2.4\times 10^{4}$ IU/mL, $p=7.7\times 10^{-8}$).

We also systematically tested for a covariate effect of HBeAg status on all our model parameters and found that it is a significant covariate on three model parameters, β, α, and δ (Table 2). Infected hepatocytes typically harbor higher cccDNA concentrations in HBeAg-positive infections [65], which provides a biological mechanism underlying the increased encapsidated pgRNA production rate, α. Further, as HBeAg-positive infections are typically linked to immune tolerance, the higher death rate of infected hepatocytes, δ, during HBeAg-negative infections may indicate a stronger antiviral immune response in these individuals [66, 67]. In S1 Text, we calculate the basic reproduction number of our model as


$${ℛ}_{0}=\frac{\lambda \;\alpha \;\beta \;\pi \;\rho }{d_{T}\;c\;\delta \;\psi_{1}\psi_{2}},$$

with $\psi_{1}=\delta +\pi +\rho =\mu$ and $\psi_{2}=\delta +\rho +\mu$.

Although the estimated infection rate (β) is lower in the HBeAg-positive group (Table 2), we found that ${ℛ}_{0}$ is larger for HBeAg-positive participants (${ℛ}_{0}=22.9$) than for HBeAg-negative participants (${ℛ}_{0}=15.0$). As the infection rate, β, is directly proportional to the basic reproduction number, this result may initially seem counter-intuitive. However, the significantly faster production rate of encapsidated pgRNA, α, and the lower death rate of infected cells, δ, in HBeAg-positive participants counterbalances the lower infection rate and results in a larger ${ℛ}_{0}$ estimate for the HBeAg-positive participants.

As mentioned above, HBeAg-positive infections tend to lead to higher viral loads. Our model also predicts increased levels in the mean predicted pre-treatment steady-states for the concentrations of infected hepatocytes, intracellular pgRNA and rcDNA, and HBV RNA and DNA for HBeAg-positive participants. We show the distribution of individual pre-treatment steady-states in Fig M of S1 Text.

### Analytical solution of the viral dynamic model identifies mechanisms driving HBV DNA and HBV RNA decay

Following treatment initiation, both HBV RNA and HBV DNA concentrations exhibited mostly biphasic declines. The rapid first phase of decline is characterized by a half-life of approximately 17 hours for both HBV RNA and HBV DNA, where this half-life is determined by $\log_{e}(2)/c$. This phase of rapid decline lasts for roughly 7 days in both HBeAg-positive and HBeAg-negative infections. On the other hand, the second, slower phase of decline differed between HBeAg-positive and HBeAg-negative individuals, with estimated half-lives of 28 and 10 days, respectively. This second phase of decline is determined by $\log_{e}(2)/\delta$.

Under the assumption that HBV DNA concentrations fall sufficiently rapidly during vebicorvir treatment to neglect *de novo* cell infections during treatment, we solved the multiscale model Eqs (6)–(12) analytically in S1 Text. We evaluated this approximation by comparing the predicted HBV DNA dynamics obtained by simulating the full ODE model Eqs (6)–(12) and the approximation obtained by neglecting new infections for each participant in Figs A and B of S1 Text for HBV DNA and RNA, respectively. The difference between the approximate and full model predictions is less than $0.3\log_{10}$ for all participants during the 28 day treatment period in this trial. However, the difference between the exact and approximate solutions are generally less than $0.1\log_{10}$ when the drug effectiveness is high, i.e., for the 200 mg and 300 mg doses.

The analytical solution for HBV RNA concentrations after the start of treatment, derived in Eq (S17) of S1 Text, is the sum of three exponentially decaying terms with rates $\psi_{1}$, *c*, and δ, whereas the concentration of HBV DNA is the sum of four exponentially decaying terms with rates $\psi_{1},\psi_{2},c$, and δ as given in Eq (S18) of S1 Text (see also Fig H of S1 Text for an illustration of these multi-exponential dynamics).

**Fig 4:**
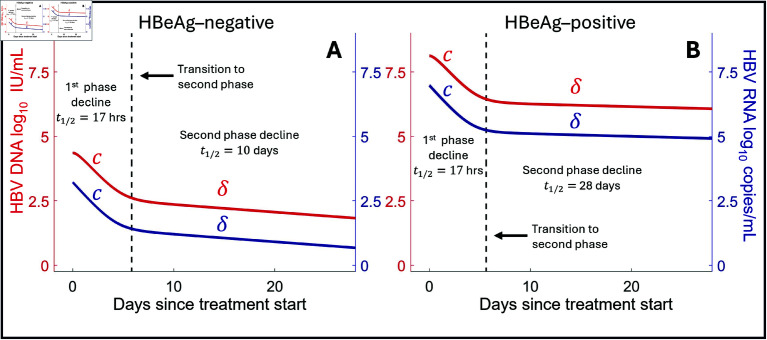
Biphasic decay of HBV RNA and HBV DNA during vebicorvir treatment Panels A and B show the HBV DNA (red) and HBV RNA (blue) decay profiles during 300 mg daily vebicorvir treatment for the population parameter estimates for HBeAg-negative and positive participants, respectively.

We also show the predicted population HBV RNA and HBV DNA decay curves in Fig 4 to illustrate the transition between the decay at rate *c* and at rate δ. These exponential decays are directly related to the mechanism of action of vebicorvir. As vebicorvir inhibits the encapsidation, and thus production of encapsidated pgRNA, the intracellular encapsidated pgRNA concentration declines with rate $\psi_{1}$ due to degradation, secretion, and reverse transcription, and very rapidly reaches a treated quasi-equilibrium level $P_{treat}^{*}$ within infected hepatocytes. This decay, at rate $\psi_{1},$ which occurs for the intracellular concentrations of both pgRNA and rcDNA is sufficiently fast to not be visible in the data. There is a corresponding decline to a treated quasi-equilibrium $C_{treat}^{*}$ with rate $\psi_{2}$ in intracellular rcDNA concentrations, and this is observable as the slight delay between treatment initation and the decay of HBV DNA at rate *c* (see red line in Fig 4).

Then, as $\rho P_{treat}^{*}<\rho P_{0}$ and $\rho C_{treat}^{*}<\rho C_{0}$, there is a corresponding fall in the secretion of HBV RNA and HBV DNA. Recalling that the system was at steady-state prior to treatment with $\rho P_{0}=cR_{0}$ and $\rho C_{0}=cV_{0}$, the rapid convergence to the treated quasi-equilibria, $P_{treat}^{*}$ and $C_{treat}^{*}$, for intracellular pgRNA and rcDNA implies that the subsequent observable HBV RNA and DNA dynamics are initially dominated by clearance, with rate *c*, during the initial phase of decline following treatment initiation. Then, due to the significant decrease of HBV DNA during the first phase of decline, the virus is not able to maintain the infected hepatocyte population at the pre-treatment level via secondary infections. Thus, the death of infected cells drives the second phase of decline and leads to viral decline at the death rate of infected hepatocytes.

For both HBeAg-positive and negative participants, intracellular pgRNA declines to the treated quasi-equilibria with a half-life of $\log_{e}(2)/\psi_{1}=0.003$ days, while intracelluar rcDNA declines to its treated equilibrium with a half-life of $\log_{e}(2)/\psi_{2}=0.27$ days. The impact of these declines on serum HBV RNA and DNA levels could potentially inform an improved understanding of the intracellular HBV life cycle. For example, by measuring the number of rcDNA copies per infected hepatocyte from a pre-treatment liver biopsy and estimating the percentage of infected hepatocytes, we could estimate the baseline total concentration of rcDNA, *C*_0_. Then, assuming that we were able to observe $\psi_{2}$, recalling that the HBV RNA and DNA declines directly inform *c* and δ, and that $V_{0}$ is typically measured in clinical studies, we find an explicit expression for the intracellular rcDNA decay rate as follows. At the quasi-steady state $cV_{0}=\rho C_{0}$, so $cV_{0}/C_{0}=\rho =\psi_{2}-\delta -\mu_{v}$ or


$$\mu_{v}=\psi_{2}-\delta -\frac{cV_{0}}{C_{0}}.$$

This relationship further demonstrates how, under appropriate circumstances, our multiscale modeling framework can facilitate the identification of intracellular mechanisms directly from clinical data.

### The first phase decay of HBV RNA is more sensitive than that of HBV DNA to CAM effectiveness

Due to the mechanism of action of CAMs in blocking intracellular pgRNA production, HBV RNA dynamics are a potential direct biomarker of target engagement, and thus, drug effectiveness [5, 68]. Here, we use our mathematical model to understand the relationship between CAM efficacy and the observed decay in HBV RNA and HBV DNA.

We performed an analytical sensitivity analysis of the response of HBV RNA and DNA to an increase in CAM effectiveness. Under the assumption that CAM treatment was sufficiently potent to neglect new infections and that the first phase of decline is sufficiently short to neglect the death of previously infected cells, we solved the multiscale model Eqs (6)–(12). In S1 Text, we used this analytical solution to evaluate the impact of parameter changes on model predictions and show that perturbations of CAM effectiveness result in larger relative changes in HBV RNA concentrations than in HBV DNA concentrations. Specifically, we show


$$\left|\frac{\partial \frac{R(t,\varepsilon )}{R_{0}}}{\partial \varepsilon }\right|⩾\left|\frac{\partial \frac{V(t,\varepsilon )}{V_{0}}}{\partial \varepsilon }\right|,$$

where *R*_0_ and $V_{0}$ are the pre-treatment steady-state HBV RNA and HBV DNA levels, respectively, and ε is the drug effectiveness. This analytical result indicates that HBV RNA, rather than HBV DNA imparts the most information regarding CAM efficacy, as has been suggested recently [37].

**Fig 5:**
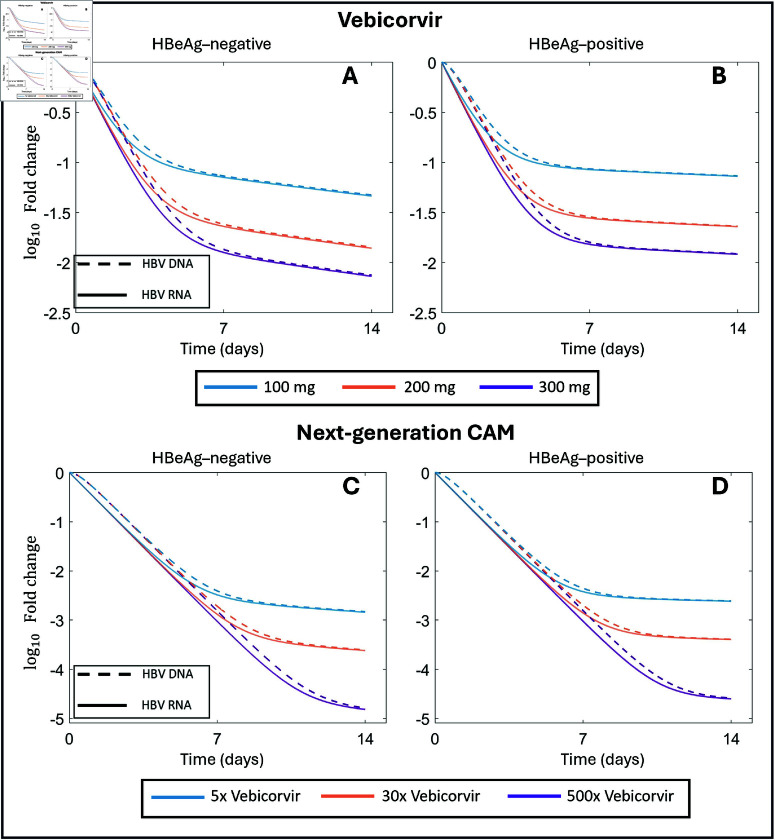
Larger fold decay in HBV RNA than HBV DNA particularly during the first phase of decline after treatment. Panels A and B show the fold decay in HBV RNA and HBV DNA during 14 days of treatment for 100 mg, 200 mg, and 300 mg of vebicorvir for the population parameter estimates for HBeAg-negative and positive participants, respectively. Panels C and D show the predicted fold decay in HBV RNA and HBV DNA during 14 days of treatments with a hypothetical next-generation CAM that is 5, 30, or 500 fold more effective at blocking the production of encapsidated pgRNA than daily administration of 300 mg of vebicorvir.

In Fig 5, we compare the predicted relative changes in $\log_{10}$ concentrations of HBV RNA and HBV DNA during the first 14 days of treatment with vebicorvir. We use the mean population parameter estimates for HBeAg-negative and HBeAg-positive participants as the viral dynamics parameters and the population estimates for 100, 200, or 300 mg of vebicorvir for the values of ε in Panels A and B, respectively. In all cases, we see that the predicted fold decline in HBV RNA concentration is larger than the corresponding prediction in HBV DNA, in agreement with our analytical results. In Fig E of S1 Text, we show the same comparison for each participant in the vebicorvir trial. In all cases, while the decay dynamics of HBV RNA and HBV DNA are similar, HBV RNA undergoes a larger relative decay, particularly in the first phase of decline where the effect of the drug is most pronounced, as shown in Fig 5.

We expect next-generation CAMs to more potently inhibit the production of encapsidated pgRNA than vebicorvir. For example, later-generation CAMs demonstrate 5-500 fold increases in *in vitro* potency [81]. Using our model and the population parameter estimates, we therefore simulated potential HBV RNA and HBV DNA dynamics following treatment with hypothetical next-generation CAM that is 5, 30, or 500 times more potent against the production of encapsidated pgRNA than the 300 mg daily dose of vebicorvir. These hypothetical CAMs have predicted effectiveness of ε=0.997,0.9995, and 0.99997, respectively. In Fig 5C and 5D , we show the predicted fold decline in HBV RNA and HBV DNA. As before, the predicted fold decline is larger in HBV RNA than HBV DNA and this difference extends throughout the first phase of decline. As the CAM effect is most pronounced during first phase of decline, it is unsurprising that the first phase of decline is predicted to have a longer duration during treatment with these next-generation CAMs.

The larger response of HBV RNA to changes in CAM effectiveness indicates that the dynamics of HBV RNA are more sensitive to treatment with a CAM than HBV DNA during the first phase of decline. As previously mentioned, this first phase of decline corresponds directly to the CAM mediated blocking of encapsidated pgRNA production. Following the initiation of treatment and on time-scales where infected cell death is negligible, intracellular encapsidated pgRNA and rcDNA amounts rapidly decay to a treated quasi-steady state. The crux of our analytical sensitivity analysis is tying the decay dynamics of these intracellular quantities to the extracellular dynamics of HBV RNA and HBV DNA. Encapsidated pgRNA approaches the treated quasi-equilibrium concentration much faster than rcDNA. Consequently, the HBV RNA dynamics will reflect the effect of CAM treatment more rapidly than HBV DNA. However, once these intracellular quantities reach their treated quasi-steady states, the dynamics of the HBV RNA and HBV DNA are similar. Thus, it is not surprising that the differences in the dynamics of HBV RNA and HBV DNA are most pronounced during the first phase of treatment mediated decline. Indeed, at the first day post-treatment initiation for all three doses of vebicorvir, we calculate


$$\left|\frac{\partial \frac{R(1,\varepsilon )}{R_{0}}}{\partial \varepsilon }\right|>1.4\left|\frac{\partial \frac{V(1,\varepsilon )}{V_{0}}}{\partial \varepsilon }\right|,$$

for both HBeAg-positive and negative participants. Accordingly, considering the early relative dynamics of HBV RNA may facilitate estimation of ε in on-going CAM monotherapy trials. However, as can be observed in Fig 5, the dynamics of the relative HBV RNA and HBV DNA concentrations are similar during the second week of treatment decline, as intracellular encapsidated pgRNA and rcDNA populations have reached their respective treated quasi-steady states by the second phase of decline. Altogether, the clinical interpretation of our result indicates that the additional utility of HBV RNA, compared to HBV DNA, in reflecting the most information regarding CAM effectiveness in preventing encapsidation is limited to the first decay phase as this phase reflects the intracellular dynamics and the mechanism of action of the CAM. As the duration of this first phase of decline is predicted to be longer during treatment with more potent next-generation CAMs (Fig 5), our results suggest that HBV RNA dynamics may be increasingly useful indicators of treatment effectiveness in on-going clinical trials.

## Discussion

Many CAMs, including the first-generation agent vebicorvir, have entered clinical trials and represent a promising treatment option for CHB. Here, we developed a multiscale model of CHB that bridges the intracellular viral life cycle and extracellular viral dynamics to understand the observed viral kinetics in a multiple ascending dose study of vebicorvir. Our model builds on the multiscale model of CHB developed by Gonçalves et al. [39] to include both the dynamics of ALT and uninfected hepatocytes. While multiscale models have been used in modeling chronic hepatitis C infection [20, 21, 25–27] and are beginning to be developed for CHB [39, 69–71], many previous modeling studies of CHB have not included the intracellular viral life cycle [32, 33, 46, 72, 74, 75]. Multiscale models, such as the model presented in this work or developed elsewhere [39, 70], offer unique insight into the intracellular and extracellular dynamics of HBV via the ability to explicitly model distinct mechanisms of action for novel small-molecule antiviral therapies and simulate the potential antiviral effects of next-generation therapies.

We fit our model to longitudinal HBV RNA, HBV DNA and ALT data of 29 individuals with chronic HBV infection treated with vebicorvir [4]. Our model describes these dynamics well both during treatment and following treatment cessation. Vebicorvir treatment led to two observable phases of decline in HBV RNA and, after a slight delay, HBV DNA. This first phase of decline was rapid in both HBV RNA and HBV DNA, with a half-life of approximately 17 hours. Our analysis of the multiscale model indicates that this phase of decline is dominated by the clearance from the circulation of both HBV RNA and HBV DNA. Our estimated clearance rate, *c*, is consistent with earlier results from Nowak et al. [29], Tsiang et al. [30] and Ribeiro et al. [32], but is much smaller than the estimate reported by Gonçalves et al. [39]. They reported *c* = 20/day, with similar model fits obtained for *c* = 5/day and *c* = 10/day. However, the rapid decay of HBV RNA and HBV DNA predicted by large values of c⩾5/day is incompatible with our viral dynamics data from baseline and day 1 post-treatment initiation. Indeed, our fitting and exploration of parameter space indicated a strong preference for c⩽5/day. We note that the viral dynamics data considered by Gonçalves et al. [39] did not include HBV RNA measurements taken before day 7 post-treatment initiation, which may explain the differences in our estimates of *c*, although Gonçalves et al. [39] suggest that the pharmacokinetics of RG7907 may play a role. The second phase of decline of HBV RNA and HBV DNA was slower and our model analysis shows it is mainly driven by the death of infected hepatocytes. We also found that vebicorvir exhibits dose-dependent efficacy, with 300 mg daily dosing leading to the highest suppression of both HBV RNA and HBV DNA. Unlike the modeling of the CAM RG7907 [39], our results do not indicate a HBeAg-dependent difference in drug efficacy. However, we identified significant HBeAg status dependent differences in the infection rate and death rate of infected hepatocytes, δ, with higher values found in HBeAg-negative participants, possibly due to the loss of immune tolerance in these participants [66]. Despite the estimated higher infection rate, β, in HBeAg-negative than in HBeAg-positive infection, we found that the significantly larger production rates of intracellular encapsidated pgRNA, α, results in a larger basic reproduction number in HBeAg-positive infection, which is consistent with the higher viral load in HBeAg-positive infection. While we were able to directly link these parameters to observable dynamics in HBV RNA and HBV DNA, we have not identified a mechanistic basis for the estimated three order of magnitude difference in β between HBeAg-positive and HBeAg-negative individuals.

Recently, there has been increased interest in using HBV RNA as a potential biomarker of treatment efficacy for CHB [38, 64]. As HBV RNA is a direct downstream product of cccDNA activity, via the production of encapisdated pgRNA, that is not directly impacted by treatment with NAs, decays in HBV RNA during NA treatment have been suggested to correspond to decays in cccDNA activity [38]. In particular, HBV RNA has been shown to predict viral rebound following treatment interruption in individuals treated with NAs [64]. Here, we evaluated HBV RNA as a indicator of CAM efficacy using our multiscale model. Specifically, we performed an analytic sensitivity analysis of our multiscale model and showed that HBV RNA concentrations are more sensitive to increases in CAM efficacy than HBV DNA concentrations during first phase decay. Our ability to distinguish between the HBV RNA and HBV DNA response to vebicorvir treatment crucially depends on our multiscale model explicitly including the dynamics of intracellular encapsidated pgRNA and rcDNA. Our analytical and simulation results suggest the continued use of HBV RNA as an important biomarker for CAM efficacy. Further, our modeling suggests that HBV RNA dynamics impart more information regarding CAM effectiveness than HBV DNA dynamics during the first phase of decline, and suggests the utility of this biomarker in on-going CAM trials. Consequently, our results highlight the potential benefits of more sensitive HBV RNA assays and of obtaining more frequent HBV RNA measurements early after treatment initiation.

Our modeling has some limitations. We did not include a mechanistic pharmacokinetic model to drive vebicorvir dynamics but rather assumed that vebicorvir concentrations rapidly reach their steady-state value during daily dosing. Consequently, we used a phenomenological model to capture vebicorvir washout and the resulting decline in CAM efficacy following treatment cessation. Using this model and data obtained after cessation of therapy, we estimated a half-life of roughly 7.3 hours for the antiviral effect of vebicorvir, which is shorter than the estimated circulating half-life of 23.5-28.4 hours for vebicorvir observed by Yuen et al. [4]. This discrepancy in estimates may be due to the infrequent sampling after the end of therapy, which led to a very large percent relative standard error in our estimate of the drug washout rate *k*. Further, our multiscale model simplified the intracellular life cycle and extracellular dynamics of HBV infection. At the intracellular level, we neglected cccDNA dynamics and potential rcDNA recycling within an infected cell, as the half-life of cccDNA has been estimated as approximately 40 days [76] and between 6.9 and 21.7 weeks in a more recent study[77], and vebicorvir has not been shown to inhibit rcDNA recycling [78]. However, as next-generation CAMs have demonstrated inhibition of rcDNA recycling, explicitly including cccDNA dynamics is a natural extension of our model. During treatment with these next-generation CAMs, we anticipate that the final phase of decline of HBV RNA and HBV DNA will correspond to the loss of infected cells either due to death or degradation of intracellular cccDNA, while ALT dynamics correspond only to the death of infected cells. Consequently, extending our modelling to these next-generation CAMs may inform the rate of cccDNA degradation *in vivo*. Furthermore, we did not model the dynamics of unencapsidated pgRNA nor the dynamics of other known HBV biomarkers, such as HB surface antigen and core-related antigen, which are useful in diagnosing CHB [79, 80], although an extension of our multiscale model could explicitly model the dynamics of these biomarkers.

All told, we developed a multiscale viral dynamic model to investigate the effect of vebicorvir monotherapy on the dynamics of HBV RNA and HBV DNA in chronically infected individuals. We note that, by including the intracellular dynamics of encapsidated pgRNA and rcDNA, our multiscale model can be used to study the effect of both NA and CAM plus NA combination therapy and future studies may include studying the effect of combination therapies on HBV dynamics. Here, we identified mechanistic differences between participants with HBeAg-positive and HBeAg-negative infection, showed that HBV RNA is more sensitive to CAM efficacy through an analytical study of our model, and finally predicted the time-scales on which HBV RNA dynamics are a potentially informative indicator of CAM efficacy.

## Supporting information

S1 Text Supporting information: A multiscale model of the action of a capsid assembly modulator for the treatment of chronic hepatitis B. (PDF)

S1 Dataset Supporting information: Viral load data. (CSV)
