# Supplementary material for: A multiscale model of the action of a capsid assembly modulator for the treatment of chronic hepatitis B
Source: PLoS Comput Biol. 2025 May 6;21(5):e1012322. doi: 10.1371/journal.pcbi.1012322 (PMC12121926; doi:10.1371/journal.pcbi.1012322)
Supplement: S1 Text — (PDF) [file pcbi.1012322.s001.pdf]

S1 Text: A multiscale model of the action of a capsid assembly modulator for the treatment of chronic hepatitis B

Sarafa A. Iyaniwura[1†], Tyler Cassidy[2†], Ruy M. Ribeiro[1], and Alan S. Perelson[1,*]

1 Theoretical Biology and Biophysics, Theoretical Division, Los Alamos National Laboratory, Los Alamos, NM 87545, USA.

2 School of Mathematics, University of Leeds, Leeds, LS2 9JT, United Kingdom

† Authors contributed equally.

* Correspondance: asp@lanl.gov

Here, we detail our analysis of the mathematical model presented in the main text. For convenience, we recall the ODE model corresponding to pre-treatment

$$\left.\begin{aligned}
\frac{\mathrm{d}T}{\mathrm{d}t} &= \lambda - \beta V T - d_T T,\\
\frac{\mathrm{d}I}{\mathrm{d}t} &= \beta V T - \delta I,\\
\frac{\mathrm{d}P}{\mathrm{d}t} &= \alpha I - (\mu_r + \delta + \pi + \rho_r) P,\\
\frac{\mathrm{d}C}{\mathrm{d}t} &= \pi P - (\mu_v + \delta + \rho_v) C,\\
\frac{\mathrm{d}R}{\mathrm{d}t} &= \rho_r P - c_r R,\\
\frac{\mathrm{d}V}{\mathrm{d}t} &= \rho_v C - c_v V,\\
\frac{\mathrm{d}A}{\mathrm{d}t} &= s + \alpha_A \delta I - c_A A.
\end{aligned}\right\} \tag{S1}$$

and during capsid assembly modulator (CAM) treatment

$$\left.\begin{aligned}
\frac{\mathrm{d}T}{\mathrm{d}t} &= \lambda - \beta VT - d_T T,\\
\frac{\mathrm{d}I}{\mathrm{d}t} &= \beta VT - \delta I,\\
\frac{\mathrm{d}P}{\mathrm{d}t} &= \alpha(1-\varepsilon(t))I - (\mu_r + \delta + \pi + \rho_r)P,\\
\frac{\mathrm{d}C}{\mathrm{d}t} &= \pi P - (\mu_v + \delta + \rho_v)\, C,\\
\frac{\mathrm{d}R}{\mathrm{d}t} &= \rho_r P - c_r R,\\
\frac{\mathrm{d}V}{\mathrm{d}t} &= \rho_v\, C - c_v V,\\
\frac{\mathrm{d}A}{\mathrm{d}t} &= s + \alpha_A\, \delta I - c_A A,
\end{aligned}\right\} \tag{S2}$$

where the drug efficacy, $\varepsilon(t)$, is defined by

$$\varepsilon(t) = \begin{cases} \varepsilon & t \leqslant \tau\,; \\ \frac{\varepsilon\, e^{-k(t-\tau)}}{\varepsilon\left(e^{-k(t-\tau)}-1\right)+1} & t > \tau. \end{cases} \tag{S3}$$

Pre-treatment steady-states

We are interested in understanding the effect of treatment with the CAM vebicorvir in 29 individuals with chronic hepatitis B virus (HBV) infection. We assume that the viral dynamics of these individuals were at steady-state at treatment initiation, and derive the infected steady-state of the pre-treatment model Eq. (S1) as the initial conditions for the treatment model Eq. (S2).

The pre-treatment model Eq. (S1) has two steady-states: an uninfected steady-state (UI_{ss}) and an infected steady-state (IF_{ss}). The UI_{ss} is given by

$$\left(T^*, I^*, P^*, C^*, R^*, V^*, A^*\right) = \left(\frac{\lambda}{d_T}, 0, 0, 0, 0, 0, \frac{s}{c_A}\right), \tag{S4}$$

where λ and d_T are the production and death rates of target cells (uninfected hepatocytes), respectively, s is the baseline rate of production of ALT and c_A is the degradation rate of ALT. The IF_{ss} $\left(T_0, I_0, P_0, C_0, R_0, V_0, A_0\right)$ is

given by

$$
\begin{aligned}
T_0 &= \frac{c_v}{\beta\rho_v\,\mathcal{M}},\\
I_0 &= \frac{\lambda}{\delta} - \frac{c_v\,d_T}{\beta\,\delta\,\rho_v\,\mathcal{M}},\\
P_0 &= \frac{\alpha}{\psi_1}\left(\frac{\lambda}{\delta} - \frac{c_v\,d_T}{\beta\,\delta\,\rho_v\,\mathcal{M}}\right),\\
C_0 &= \lambda\,\mathcal{M} - \frac{c_v\,d_T}{\beta\,\rho_v},\\
R_0 &= \frac{\alpha\rho_r}{c_r\,\psi_1}\left(\frac{\lambda}{\delta} - \frac{c_v\,d_T}{\beta\,\delta\,\rho_v\,\mathcal{M}}\right),\\
V_0 &= \frac{\lambda\,\rho_v\,\mathcal{M}}{c_v} - \frac{d_T}{\beta},\\
A_0 &= \frac{c}{c_A} + \frac{\alpha_A}{c_A}\left(\lambda - \frac{c_v\,d_T}{\beta\,\rho_v\,\mathcal{M}}\right),
\end{aligned}
\tag{S5}
$$

where $\mathcal{M} = \pi\,\alpha/(\delta\,\psi_1\,\psi_2)$, with $\psi_1 = \mu_r + \delta + \pi + \rho_r$ and $\psi_2 = \mu_v + \delta + \rho_v$. Note that I_0, P_0, C_0, R_0 and A_0 can be written in terms of the steady-state HBV DNA concentration (V_0) as follows

$$
\begin{aligned}
&I_0 = \frac{c_v}{\delta\,\rho_v\,\mathcal{M}}V_0, \qquad P_0 = \frac{\alpha\,c_v}{\delta\,\rho_v\,\mathcal{M}(\mu_r+\delta+\pi+\rho_r)}V_0, \qquad R_0 = \frac{\alpha\,c_v\,\rho_r}{\delta\,c_r\,\rho_v\,\mathcal{M}(\mu_r+\delta+\pi+\rho_r)}V_0,\\
&C_0 = \frac{c_v}{\rho_v}V_0, \qquad A_0 = \frac{1}{c_A}\left(s + \frac{\alpha_A\,c_v}{\rho_v\,\mathcal{M}}V_0\right).
\end{aligned}
\tag{S6}
$$

Basic reproduction number

Since we are interested in the strictly positive steady-states of Eq. (S1) corresponding to chronic infection, we derive a condition that guarantees that the expressions in Eq. (S5) are positive. Using the next-generation matrix approach [1, 2], we compute the basic reproduction number ($\mathcal{R}_0$) of the pre-treatment model Eq. (S1). To do this, we consider the equations for infected cells (I), intracellular pgRNA (P) and rcDNA (C), HBV RNA (R) and HBV DNA (V) in Eq. (S1), given by

$$\left.\begin{aligned}
\frac{\mathrm{d}I}{\mathrm{d}t} &= \beta VT - \delta I,\\
\frac{\mathrm{d}P}{\mathrm{d}t} &= \alpha I - (\mu_r + \delta + \pi \ + \rho_r)P,\\
\frac{\mathrm{d}C}{\mathrm{d}t} &= \pi\, P - (\mu_v + \delta + \rho_v)\, C,\\
\frac{\mathrm{d}R}{\mathrm{d}t} &= \rho_r P - c_r R,\\
\frac{\mathrm{d}V}{\mathrm{d}t} &= \rho_v\, C - c_v V.
\end{aligned}\right\} \tag{S7}$$

At the uninfected steady-state (UI_{ss}) given in Eq. (S4), we construct the Jacobian matrix $\mathcal{F}$ for new target cell infection and matrix $\mathcal{V}$ describing all other transitions within the sub-model in Eq. (S7). These matrices are given by

$$\mathcal{F} = \begin{pmatrix} 0 & 0 & 0 & 0 & \frac{\lambda\beta}{d_T} \\ 0 & 0 & 0 & 0 & 0 \\ 0 & 0 & 0 & 0 & 0 \\ 0 & 0 & 0 & 0 & 0 \\ 0 & 0 & 0 & 0 & 0 \end{pmatrix}, \qquad \mathcal{V} = \begin{pmatrix} \delta & 0 & 0 & 0 & 0 \\ -\alpha & \psi_1 & 0 & 0 & 0 \\ 0 & -\pi & \psi_2 & 0 & 0 \\ 0 & -\rho_r & 0 & c_r & 0 \\ 0 & 0 & -\rho_v & 0 & c_v \end{pmatrix}. \tag{S8}$$

where $\psi_1 = \mu_r + \delta + \pi + \rho_r$ and $\psi_2 = \mu_v + \delta + \rho_v$. The next-generation matrix is defined as $M = \mathcal{F}\mathcal{V}^{-1}$, and its largest eigenvalue is our desired basic reproduction number, and it is given by

$$\mathcal{R}_0 = \frac{\lambda\, \alpha\, \beta\, \pi\, \rho_v}{d_T\, c_v\, \delta\, \psi_1 \psi_2}, \tag{S9}$$

where ψ_1 and ψ_2 are as defined in Eq. (S8). The steady-state HBV DNA concentration (V_0) can be written in terms of $\mathcal{R}_0$ as

$$V_0 = \frac{d_T}{\beta}\big(\mathcal{R}_0 - 1\big). \tag{S10}$$

Immediately, we see that $V_0 > 0$ only if $\mathcal{R}_0 > 1$, while the infected and uninfected steady-states coalesce when $\mathcal{R}_0 = 1$. Numerical simulation indicates an exchange of stability when these steady-states coalesce, which suggest the presence of a transcritical bifurcation at $\mathcal{R}_0 = 1$.

The numerator of $\mathcal{R}_0$ in Eq. (S9) is the product of the target cell production rate (λ), the encapsidated pgRNA production rate (α), the reverse transcription rate of encapsidated pgRNA to rcDNA (π), the virus infectious rate (β), and the rate that rcDNA is packaged into virions and secreted into the circulation (ρ_v). All these parameters contribute to virus production. On the other hand, the denominator is the product of the target cells death rate (d_T), death rate of infected cells (δ), clearance rate of HBV DNA (c_v), and the parameters ψ_1 and ψ_2, which are given

above. Most of the rates in the denominator contribute to virus loss. Therefore, when $\mathcal{R}_0 > 1$, we have

$$\lambda\,\beta\,\alpha\,\pi\rho_v\ > d_T\,c_v\,\delta\psi_1\psi_2, \tag{S11}$$

which implies that the product of the rates that contribute to virus production must be greater than the product of the rates that contribute to the virus loss for a positive infected steady-state Eq. (S6) to exist.

Analytical solution of reduced ODE system

To study the theoretical decay profiles of HBV DNA and HBV RNA during treatment with a CAM we assume that treatment with vebicorvir is sufficiently potent to neglect new infections during treatment [3–5]. We thus set $\beta = 0$ in the multiscale model Eq. (S2) to obtain the following reduced system of ODEs:

$$\begin{aligned}
\frac{\mathrm{d}}{\mathrm{dt}}I(t) &= -\delta\,I(t),\\
\frac{\mathrm{d}}{\mathrm{dt}}P(t) &= \alpha(1-\varepsilon)\,I(t) - (\mu_r + \delta + \pi + \rho_r)\,P(t),\\
\frac{\mathrm{d}}{\mathrm{dt}}C(t) &= \pi\,P(t) - (\mu_v + \delta + \rho_v)\,C(t),\\
\frac{\mathrm{d}}{\mathrm{dt}}R(t) &= \rho_r\,P(t) - c_r\,R(t),\\
\frac{\mathrm{d}}{\mathrm{dt}}V(t) &= \rho_v\,C(t) - c_v\,V(t).
\end{aligned} \tag{S12}$$

Here, we focus on the dynamics of HBV DNA and HBV RNA and do not consider the dynamics of ALT. The reduced ODE system Eq. (S12) is linear and can be solved analytically. As before, we impose

$$I(0) = I_0, \quad P(0) = P_0, \quad C(0) = C_0, \quad R(0) = R_0, \quad \text{and} \quad V(0) = V_0, \tag{S13}$$

where I_0, P_0, C_0, R_0, and V_0 are given in Eq. (S5). We obtain

$$I(t) = I_0\,e^{-\delta t}, \qquad \text{where} \qquad I_0 = \frac{\lambda}{\delta} - \frac{c_v\,d_T\,\psi_1\,\psi_2}{\beta\,\rho_v\,\alpha\,\pi}. \tag{S14}$$

The total concentrations of intracellular pgRNA (P) and rcDNA (C) satisfy

$$P(t) = P_0\,e^{-\psi_1 t} + \frac{\alpha\,(1-\varepsilon)I_0}{(\psi_1 - \delta)}\left(e^{-\delta t} - e^{-\psi_1 t}\right), \tag{S15}$$

and

$$C(t) = C_0\, e^{-\psi_2 t} + \frac{\alpha\, \pi\, (1-\varepsilon) I_0}{(\psi_1-\delta)(\psi_2-\delta)}\, e^{-\delta t} + \frac{\pi}{(\psi_2-\psi_1)} \left[P_0 - \alpha\, I_0\, \frac{(1-\varepsilon)}{(\psi_1-\delta)}\right] \left(e^{-\psi_1 t} - e^{-\psi_2 t}\right), \tag{S16}$$

where $P_0 = \frac{\alpha\,\lambda}{\psi_1\,\delta} - \frac{c_v\, d_T\, \psi_2}{\beta\, \rho_v\, \pi}$ and $C_0 = \frac{\alpha\,\pi\,\lambda}{\delta\,\psi_1\,\psi_2} - \frac{c_v\, d_T}{\rho_v\,\beta}$.

Finally, the HBV RNA (R) and HBV DNA (V) dynamics are given by

$$R(t) = R_0\, e^{-c_r t} + \frac{\rho_r\, \alpha\, (1-\varepsilon) I_0}{(\psi_1-\delta)(c_r-\delta)}\, e^{-\delta t} + \frac{\rho_r}{(c_r-\psi_1)} \left(P_0 - \alpha\, I_0\, \frac{(1-\varepsilon)}{(\psi_1-\delta)}\right) \left(e^{-\psi_1 t} - e^{-c_r t}\right). \tag{S17}$$

and

$$\begin{aligned} V(t) = {} & V_0\, e^{-c_v t} + \frac{\rho_v\, \pi\, \alpha\, I_0\, (1-\varepsilon)}{(\psi_1-\delta)\,(\psi_2-\delta)\,(c_v-\delta)}\, e^{-\delta t} + \frac{\rho_v\, C_0}{(c_v-\psi_2)} \left(e^{-\psi_2 t} - e^{-c_v t}\right) \\ & + \frac{\rho_v\, \pi}{(\psi_2-\psi_1)(c_v-\psi_1)} \left(P_0 - \frac{\alpha\, I_0\, (1-\varepsilon)}{(\psi_1-\delta)}\right) \left(e^{-\psi_1 t} - e^{-\psi_2 t}\right) - \frac{\pi\alpha\, \rho_v\, I_0\, (1-\varepsilon)}{(\psi_1-\delta)\,(\psi_2-\delta)\,(c_v-\delta)}\, e^{-c_v t}, \end{aligned} \tag{S18}$$

where $R_0 = \frac{\alpha\,\rho_r\,\lambda}{c_r\,\psi_1\,\delta} - \frac{c_v\,\rho_r\,d_T\,\psi_2}{c_r\,\rho_v\,\beta\,\pi}$ and $V_0 = \frac{\rho_v\,\alpha\,\pi\,\lambda}{c_v\,\delta\,\psi_1\,\psi_2} - \frac{d_T}{\beta}$.

Evaluating the assumption of no new infections during treatment

To obtain our analytical results, we assumed that treatment is sufficiently potent to neglect all new infections during therapy. This assumption has proved useful in viral dynamic modeling [5, 6] and allowed us to analytically solve the viral dynamics model. However, the high baseline viral load in HBeAg-positive participants and the relatively slow clearance of virus during therapy indicates that this assumption may be overly strong in the case of HBV. We therefore compare the numerical solution of the full ODE model given by Eq. (S2) and the solution of the approximate model. Using the best-fit parameters for each participant, shown in Table A, we plot the $\log_{10}$ difference between the solutions of the approximate and full model for HBV DNA and RNA concentrations for $\varepsilon \geqslant 0.9$ in Fig A and Fig B, respectively.

The difference between the approximate and full model predictions is less than $0.3\,\log_{10}$ for all participants during the 28 days of treatment in the vebicorvir trial. Unsurprisingly, the approximate model with no post-treatment infections over-estimates the HBV DNA decay during treatment. The approximate and full models begin to diverge for treatment periods longer than the treated period of 28 days in the current trial, indicating that the simplifying assumption of no new infections following treatment initiation is not reasonable for longer durations of treatment. Given the high potency of the first-generation CAM, vebicorvir, and recently developed next-generation

Table A. Estimated individual parameters. We fixed $c = 1$/day and $\mu = 0$, and estimated $\rho = 2.48$/day and $c_A = 0.057$/day without random effects for all individuals. Also, as stated in the main text $\lambda = 5.2 \times 10^4$ cells/mL/day and $d_T = 0.004$/day were fixed parameters throughout the study.

ID	HBeAg	Dose	ε	k	β	$\mathcal{R}_0$	α	δ	π	A_{ue}	A_0
3	Negative	100 mg	0.965	2.28	2.8×10^{-7}	16.7	0.52	0.107	236.3	16.9	51.5
6	Negative	100 mg	0.907	2.30	1.1×10^{-8}	8.1	3.8	0.06	2045.5	16.0	44.7
8	Negative	200 mg	0.837	2.27	5.6×10^{-7}	5.5	0.07	0.09	49.2	33.7	30.7
9	Negative	200 mg	0.965	2.35	2.0×10^{-9}	26.1	55.2	0.053	208.3	16.3	71.8
10	Negative	200 mg	0.923	2.25	6.2×10^{-8}	10.5	1.28	0.092	101.7	21.3	43.8
12	Negative	300 mg	0.674	2.29	2.3×10^{-7}	9.15	0.24	0.075	108.7	21.8	44.0
18	Negative	100 mg	0.981	2.28	1.0×10^{-6}	8.22	0.06	0.088	220.9	14.9	16.6
19	Negative	200 mg	0.997	2.38	1.9×10^{-6}	15.7	0.04	0.052	379.9	16.9	21.3
23	Negative	100 mg	0.511	2.29	9.2×10^{-7}	12.2	0.08	0.072	242.3	109.9	22.2
26	Negative	200 mg	0.979	2.36	4.0×10^{-5}	44.5	0.004	0.05	205.9	10.7	10.4
27	Negative	300 mg	0.992	2.25	7.2×10^{-7}	10.9	0.13	0.105	562.4	25.6	30.4
29	Negative	300 mg	0.972	2.30	1.8×10^{-6}	29.3	0.11	0.082	404.6	36.7	39.1
5	Positive	200 mg	0.987	2.16	6.9×10^{-12}	6.26	4055.1	0.056	119.8	14.8	297.9
7	Positive	100 mg	0.946	2.29	2.5×10^{-11}	12.1	1024.8	0.027	120.7	20.2	61.1
11	Positive	200 mg	0.874	2.30	6.2×10^{-11}	32.5	833.2	0.02	230.3	24.0	113.8
13	Positive	300 mg	0.994	2.29	3.6×10^{-10}	26.5	150.5	0.026	158.4	19.28	41.8
15	Positive	100 mg	0.950	2.30	3.3×10^{-11}	18.3	994.2	0.023	195.2	19.8	28.5
16	Positive	100 mg	0.761	2.29	7.1×10^{-12}	11.7	3628.9	0.028	173.7	30.9	30.0
20	Positive	100 mg	0.931	2.29	8.8×10^{-11}	29.0	562.0	0.022	332.8	44.0	15.7
21	Positive	100 mg	0.871	2.31	1.9×10^{-10}	29.8	274.6	0.022	389.6	20.1	24.0
24	Positive	200 mg	0.926	2.28	4.1×10^{-10}	14.6	81.4	0.028	50.0	22.0	152.4
28	Positive	300 mg	0.964	2.30	2.4×10^{-09}	29.1	35.2	0.037	330.9	24.8	55.0
31	Positive	300 mg	0.991	2.35	8.5×10^{-10}	36.3	80.5	0.023	67.8	19.2	40.0
32	Positive	300 mg	0.995	2.32	2.2×10^{-10}	32.4	312.5	0.026	97.0	38.0	29.2
33	Positive	300 mg	0.964	2.27	9.4×10^{-10}	20.3	69.4	0.041	172.8	37.6	29.3
34	Positive	200 mg	0.985	2.32	8.1×10^{-11}	15.4	484.8	0.032	251.5	17.6	52.8
35	Positive	200 mg	0.990	2.34	7.2×10^{-11}	41.7	735.7	0.016	306.4	35.0	19.2
36	Positive	300 mg	0.998	2.34	7.9×10^{-11}	21.7	508.1	0.023	123.8	66.2	30.2
37	Positive	100 mg	0.880	2.30	1.1×10^{-11}	10.2	1764.1	0.025	334.2	12.1	34.8

CAMs, this result suggests that the assumption of no new infections is reasonable when modeling trials with a relatively short duration.

Comparison of HBV RNA and DNA dynamics during CAM treatment

We wish to measure how sensitive the treated HBV RNA and HBV DNA dynamics are to changes in CAM efficacy, ε. As before, we assume the system was at steady-state prior to treatment, but now, instead of assuming there are no new infections, we assume that the infected cell concentration is constant in a short time frame following treatment initiation.

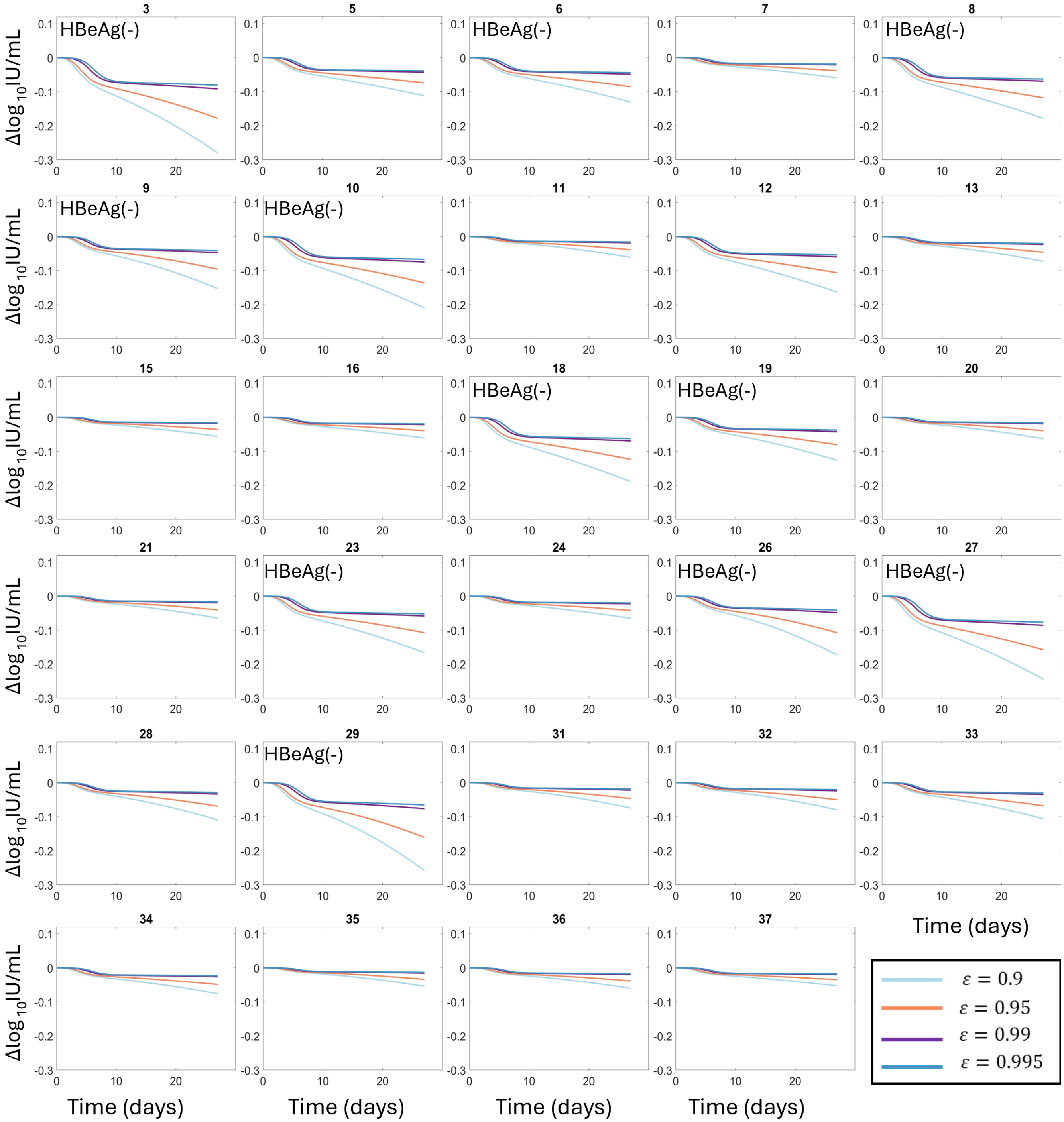

Fig A. Difference between the approximate and numerical solutions for HBV DNA neglecting new infections. A comparison between the analytical solution obtained under the assumption that $\beta = 0$ and the solution of the full model Eq. (S2) for increasing CAM efficacies, ε for all participants, with HBeAg-negative participants noted in corresponding plots. For each value of ε, the solid lines show the $\log_{10}$ difference between the predicted HBV DNA concentrations obtained by simulating the full ODE model Eq. (S2) and the analytical solution under the assumption $\beta = 0$ for 28 days of treatment. Model parameter values for generating the curves for each individual are given in Table A.

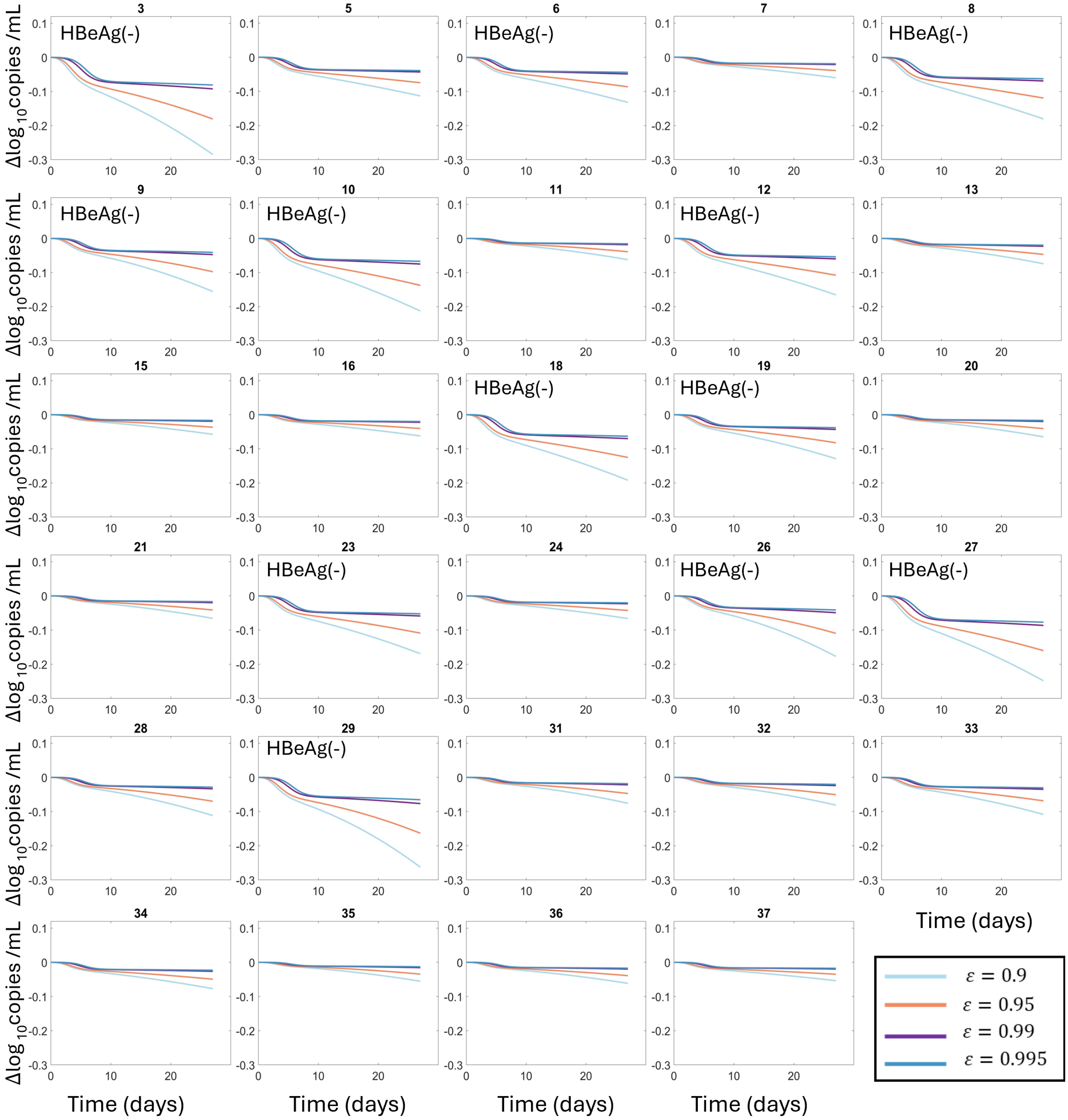

Fig B. Difference between the approximate and numerical solutions for HBV RNA neglecting new infections. A comparison between the analytical solution obtained under the assumption that $\beta = 0$ and the solution of the full model Eq. (S2) for increasing CAM efficacies, ε for all participants, with HBeAg-negative participants noted in corresponding plots. For each value of ε, the solid lines show the $\log_{10}$ difference between the predicted HBV RNA concentrations obtained by simulating the full ODE model Eq. (S2) and the analytical solution under the assumption $\beta = 0$ for 28 days of treatment. Model parameter values for generating the curves for each individual are given in Table A.

The multiscale model Eq. (S2) then reduces to

$$\left.\begin{aligned}\frac{\mathrm{d}}{\mathrm{dt}}P(t) &= \alpha I_0(1-\varepsilon) - \psi_1 P(t)\\ \frac{\mathrm{d}}{\mathrm{dt}}C(t) &= \pi P(t) - \psi_2 C(t)\\ \frac{\mathrm{d}}{\mathrm{dt}}R(t) &= \rho_r P(t) - c_r R(t)\\ \frac{\mathrm{d}}{\mathrm{dt}}V(t) &= \rho_v C(t) - c_v V(t).\end{aligned}\right\} \tag{S19}$$

While this assumption appears reasonable over the short duration of the phase I clinical trial given the long half-life of infected hepatocytes, we test the accuracy of this assumption in Figs C and D. Specifically, we show the $\log_{10}$ difference between the solution of the full model and the analytical solution of Eq. (S19) that follows the assumption that $I(t) = I_0$. The approximation is quite accurate during the first week of treatment, which corresponds to the initial phase of decline in HBV DNA and RNA. As we would expect, the approximation and true solution begin to diverge as death of infected hepatocytes begins to drive HBV DNA and RNA dynamics during the second phase of decline. Indeed, the difference between the analytical and numerical solution begins to grow linearly on the log scale, which corresponds to the exponential decay of infected hepatocytes that is neglected in the analytical solution. Furthermore, the difference between the approximate and numerical solution of the model are more pronounced for HBeAg-negative participants, which is to be expected given the significantly larger death rate of infected hepatocytes, δ, that we estimated in these participants.

Under the assumption of constant infected cells, we begin by considering the intracellular dynamics of $P(t)$ and $C(t)$. Solving for $P(t)$ in Eq. (S19) gives

$$P_\varepsilon(t) = \left[P_0 + \frac{\alpha I_0(1-\varepsilon)}{\psi_1}\left(e^{\psi_1 t} - 1\right)\right] e^{-\psi_1 t}, \tag{S20}$$

where $P_0 = (\alpha I_0)/\psi_1$ and where we explicitly write the dependence of P on the CAM efficacy ε denoting the solution $P_\varepsilon(t)$. Then, replacing $(\alpha I_0)/\psi_1$ by P_0, we obtain

$$\frac{P_\varepsilon(t)}{P_0} = \left[1 + (1-\varepsilon)\left(e^{\psi_1 t} - 1\right)\right] e^{-\psi_1 t}. \tag{S21}$$

Substituting $P_\varepsilon(t)$ into the equation for $C(t)$ in Eq. (S19) and solving the corresponding ODE, we obtain

$$C_\varepsilon(t) = \left[C_0 + \pi P_0 \int_0^t e^{(\psi_2 - \psi_1)s} + (1-\varepsilon)\left(e^{\psi_2 s} - e^{(\psi_2-\psi_1)s}\right) \mathrm{d}s\right] e^{-\psi_2 t}. \tag{S22}$$

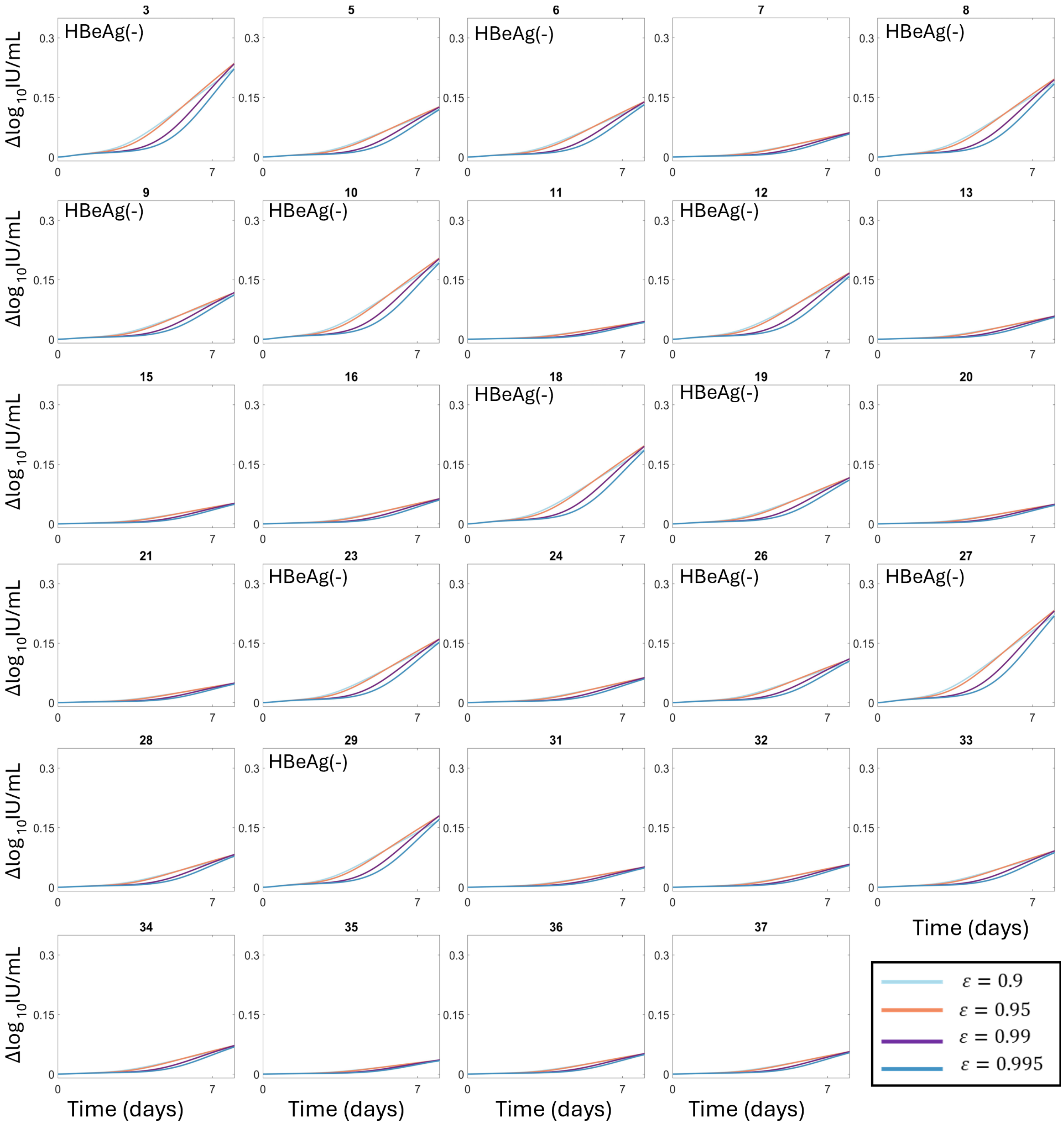

Fig C. Difference between the approximate and numerical solutions for HBV DNA for constant infected hepatocytes concentrations. A comparison between the analytical solution from the assumption that $I(t) = I_0$ and the solution of the full model Eq. (S2) for increasing CAM efficacies, ε for all participants, with HBeAg-negative participants noted in corresponding plots. For each value of ε, the solid lines show the $\log_{10}$ difference between the predicted HBV RNA concentrations obtained by simulating the full ODE model Eq. (S2) and the analytical solution under the assumption $I(t) = I_0$ for 7 days of treatment. These results were generated using the estimated individuals parameters in Table A. Model parameter values for generating the curves each individual are given in Table A.

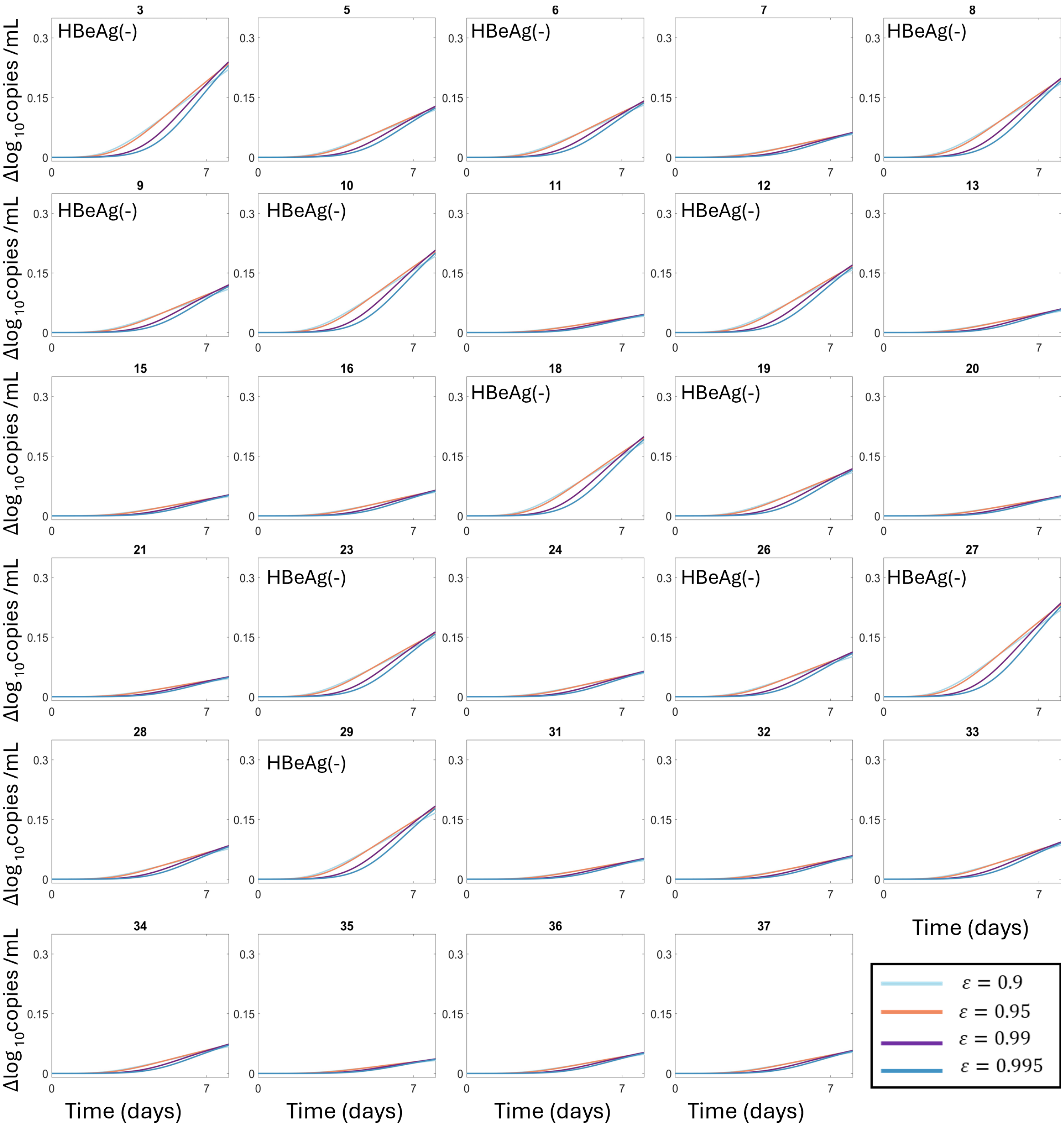

Fig D. Difference between the approximate and numerical solutions for HBV RNA for constant infected hepatocytes concentrations. A comparison between the analytical solution from the assumption that $I(t) = I_0$ and the solution of the full model Eq. (S2) for increasing CAM efficacies, ε for all participants, with HBeAg-negative participants noted in corresponding plots. For each value of ε, the solid lines show the $\log_{10}$ difference between the predicted HBV RNA concentrations obtained by simulating the full ODE model Eq. (S2) and the analytical solution under the assumption $I(t) = I_0$ for 7 days of treatment. These results were generated using the estimated individuals parameters in Table A. Model parameter values for generating the curves for each individual are given in Table A.

Imposing the initial condition $C_0 = (\pi P_0)/\psi_2$ in Eq. (S22), we obtain

$$\begin{aligned}\frac{C_\varepsilon(t)}{C_0} = \Bigg[1 + \left(\frac{\psi_2}{\psi_2 - \psi_1}\right)\left(e^{(\psi_2-\psi_1)t} - 1\right) \\ + (1-\varepsilon)\left[\left(e^{\psi_2 t} - 1\right) - \left(\frac{\psi_2}{\psi_2 - \psi_1}\right)\left(e^{(\psi_2-\psi_1)t} - 1\right)\right]\Bigg] e^{-\psi_2 t}.\end{aligned} \tag{S23}$$

To simplify notation, we write Eq. (S23) as

$$\frac{C_\varepsilon(t)}{C_0} = \Big[\sigma(t) + (1-\varepsilon)\Upsilon(t)\Big]e^{-\psi_2 t},$$

where

$$\sigma(t) = 1 + \left(\frac{\psi_2}{\psi_2 - \psi_1}\right)\left(e^{(\psi_2-\psi_1)t} - 1\right) \quad \text{and} \quad \Upsilon(t) = \left[\left(e^{\psi_2 t} - 1\right) - \left(\frac{\psi_2}{\psi_2 - \psi_1}\right)\left(e^{(\psi_2-\psi_1)t} - 1\right)\right].$$

We can thus calculate the sensitivity of the intracellular quantities $P(t)$ and $C(t)$ to CAM efficacy through

$$\frac{\partial \frac{P_\varepsilon(t)}{P_0}}{\partial \varepsilon} = -\left(1 - e^{-\psi_1 t}\right) \qquad \text{and} \qquad \frac{\partial \frac{C_\varepsilon(t)}{C_0}}{\partial \varepsilon} = -\Upsilon(t)e^{-\psi_2 t}. \tag{S24}$$

We immediately note that for fixed t,

$$\Upsilon(t) = \left(\int_0^t \psi_2 e^{\psi_2 s} - \psi_2 e^{(\psi_2-\psi_1)s}\mathrm{d}s\right)e^{-\psi_2 t} \geqslant 0.$$

Therefore, both of the quantities in Eq. (S24) decrease with respect to ε. Consequently, we find

$$\left|\frac{\partial \frac{P_\varepsilon(t)}{P_0}}{\partial \varepsilon}\right| = \left(1 - e^{-\psi_1 t}\right) \quad \text{and} \quad \left|\frac{\partial \frac{C_\varepsilon(t)}{C_0}}{\partial \varepsilon}\right| = \Upsilon(t)e^{-\psi_2 t}.$$

Since $\rho_r = \rho_v$, $\mu_r = \mu_v$, and $\pi > 0$, we have $\psi_1 > \psi_2$ which implies

$$1 - e^{-\psi_1 t} \geqslant 1 - e^{-\psi_2 t}, \tag{S25}$$

and

$$\left(\frac{\psi_2}{\psi_2 - \psi_1}\right)\left(e^{(\psi_2-\psi_1)t} - 1\right)e^{-\psi_2 t} = \psi_2 e^{-\psi_2 t}\int_0^t e^{(\psi_2-\psi_1)s}\mathrm{d}s \geqslant 0,$$

which gives

$$\left|\frac{\partial \frac{P_\varepsilon(t)}{P_0}}{\partial \varepsilon}\right| = 1 - e^{-\psi_1 t} \geqslant 1 - e^{-\psi_2 t} - \left(\frac{\psi_2}{\psi_2 - \psi_1}\right)\left(e^{(\psi_2-\psi_1)t} - 1\right) e^{-\psi_2 t} = \Upsilon(t) e^{-\psi_2 t} = \left|\frac{\partial \frac{C_\varepsilon(t)}{C_0}}{\partial \varepsilon}\right|.$$

Next, we consider the formal solutions for $R(t)$ and $V(t)$ given by

$$R(t,\varepsilon) = R_0\, e^{-ct} + \int_0^t \rho_r\, P_\varepsilon(s)\, e^{-c(t-s)}\, \mathrm{d}s \quad \text{and} \quad V(t,\varepsilon) = V_0\, e^{-ct} + \int_0^t \rho_v\, C_\varepsilon(s)\, e^{-c(t-s)}\, \mathrm{d}s,$$

where, once again, we explicitly included dependence on the treatment efficacy ε. We note that

$$\frac{R(t,\varepsilon)}{R_0} = e^{-ct} + \int_0^t \frac{\rho_r P_\varepsilon(s)}{R_0} e^{-c(t-s)}\, \mathrm{d}s \quad \text{and} \quad \frac{V(t,\varepsilon)}{V_0} = e^{-ct} + \int_0^t \frac{\rho_v C(s)}{V_0}\, e^{-c(t-s)}\, \mathrm{d}s.$$

Recalling that $R_0 = \rho_r P_0/c$ and $V_0 = \rho_v C_0/c$, we find

$$\frac{R(t,\varepsilon)}{R_0} = e^{-ct} + \int_0^t \frac{cP_\varepsilon(s)}{P_0} e^{-c(t-s)}\mathrm{d}s \quad \text{and} \quad \frac{V(t,\varepsilon)}{V_0} = e^{-ct} + \int_0^t \frac{c\,C(s)}{C_0} e^{-c(t-s)}\mathrm{d}s. \tag{S26}$$

Then, it is simple to compute

$$\frac{\partial \frac{R(t,\varepsilon)}{R_0}}{\partial \varepsilon} = \int_0^t c \frac{\partial \frac{P_\varepsilon(s)}{P_0}}{\partial \varepsilon} e^{-c(t-s)}\mathrm{d}s \quad \text{and} \quad \frac{\partial \frac{V(t,\varepsilon)}{V_0}}{\partial \varepsilon} = \int_0^t c \frac{\partial \frac{C_\varepsilon(s)}{C_0}}{\partial \varepsilon} e^{-c(t-s)}\mathrm{d}s,$$

which, since both P and C are strictly decreasing in ε, gives

$$\left|\frac{\partial \frac{R(t,\varepsilon)}{R_0}}{\partial \varepsilon}\right| - \left|\frac{\partial \frac{V(t,\varepsilon)}{V_0}}{\partial \varepsilon}\right| = \int_0^t c \left(\left|\frac{\partial \frac{P_\varepsilon(s)}{P_0}}{\partial \varepsilon}\right| - \left|\frac{\partial \frac{C_\varepsilon(s)}{C_0}}{\partial \varepsilon}\right|\right) e^{-c(t-s)}\mathrm{d}s \geqslant 0.$$

We thus conclude that the relative change in HBV RNA is larger than the corresponding changes in HBV DNA to changes in CAM efficacy during the period in which $I(t) = I_0$, i.e., the first phase of viral decline as shown in Figs C and D.

In Fig E, we show a comparison between the changes in $\log_{10}$ concentrations of HBV RNA and HBV DNA from baseline during the first 7 days of treatment with vebicorvir using the full model Eq. (S2), which confirms our approximate analytical result. We note that, while the ultimate fold decay between HBV RNA and HBV DNA are similar, HBV RNA is more sensitive to treatment with vebircorvir, as demonstrated by a larger relative decay during the first 4 days or so of treatment where the $I(t) = I_0$ holds best (Figs C and D).

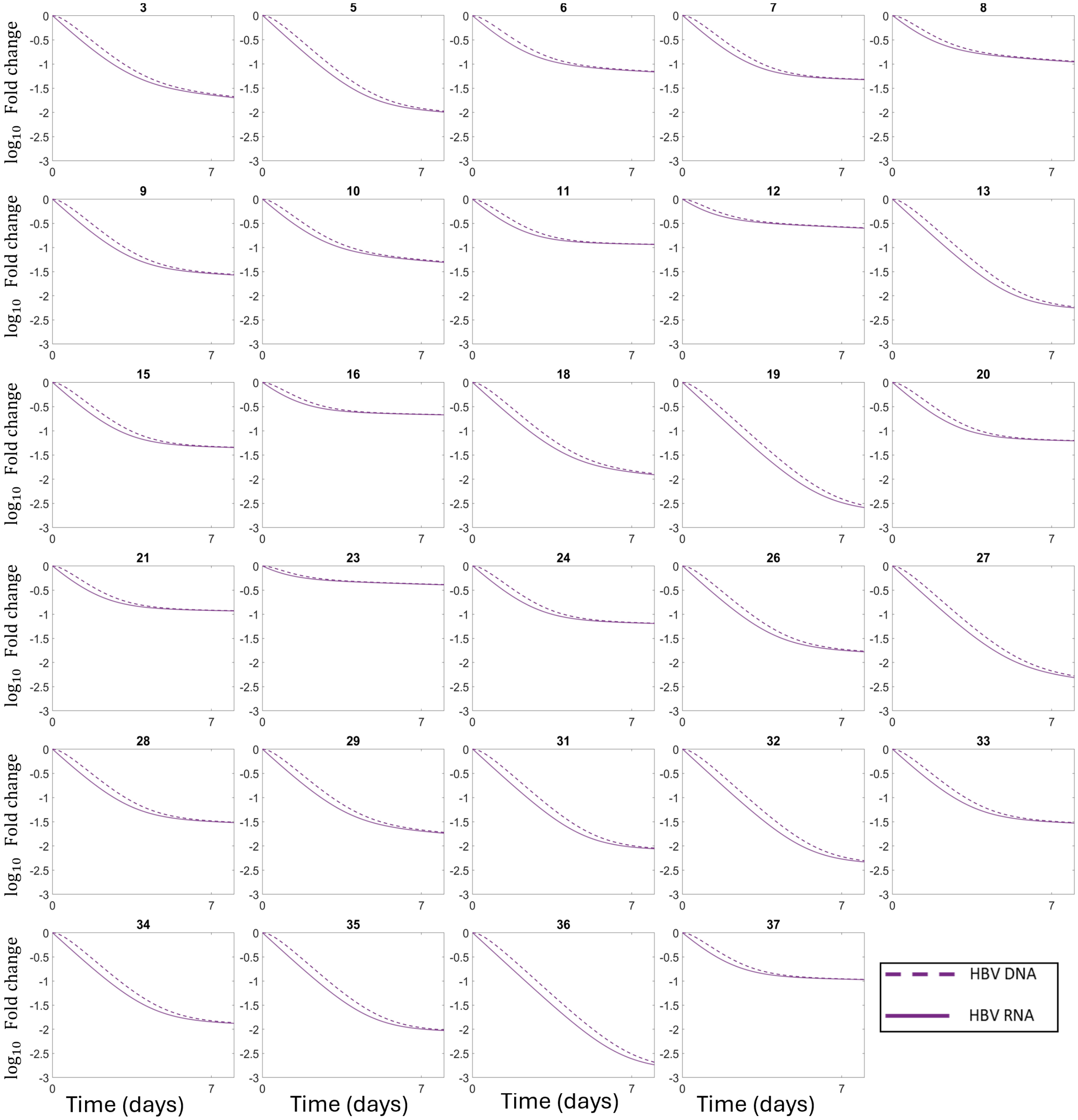

Fig E. Fold change in HBV RNA and DNA for all participants The predicted $\log_{10}$ HBV RNA and HBV DNA change from baseline trial participants. For each participant, the dashed lines show the fold change in HBV DNA concentrations while the solid lines show the fold change in HBV RNA concentrations during the first 7 days of treatment. These results were generated using the estimated individuals parameters in Table A.

Alternative model formulations

The model proposed in the main text is a multiscale model that includes the main events of the HBV lifecycle. It is also similar to models in previous studies of both HBV and hepatitis C virus (HCV) infection [5, 7–9]. Yet, specific

choices were made and alternatives parameterizations and/or structures could be proposed. In building the model and then assessing its robustness, we tested several of these assumptions and present these alternative models here in detail. We used both statistical considerations (the corrected Bayesian information criterion, BICc [10]) and biological considerations, including results from previous studies, to choose the best models.

We can divide the different models we tested into three groups, summarized in Tables B, C, and D. First, we tested sensitivity to model parameters that we kept fixed, namely c and μ, as shown in Table B. These parameters were kept fixed, rather than fit, because they are difficult to estimate with the available data. From these results, we chose $c = 1$/day and $\mu = 0$.

Table B. Sensitivity analysis on fixed model parameters. Negative log-likelihood and corrected Bayesian information criteria (BICc) for models with different values of the intracellular decay rate (μ) of encapsidated pgRNA (P) and rcDNA (C), and the extracellular clearance rate (c) of HBV RNA (R) and HBV DNA (V). This sensitivity analysis was performed based on the best model (first row) with $\mu_r = \mu_v = \mu$ and $c_v = c_r = c$ used as fixed parameters, and included covariates of drug dose for drug effectiveness and of HBeAg status for β, α, δ, random effects on all parameters except ρ, and a correlation between β and α.

Model no.	Description	Negative log-likelihood	BICc
Best model	$\mu = 0$, $c = 1$	-120.5	12.1
1	$\mu = 0.05$	-114.7	18.0
2	$\mu = 0.1$	-116.8	15.9
3	$\mu = 0.2$	-114.8	17.9
4	$\mu = 0.3$	-114.8	17.9
5	$\mu = 0.4$	-114.3	18.4
6	$\mu = 0.5$	-117.4	15.2
7	$\mu = 0.6$	-115.1	17.6
8	$\mu = 0.7$	-114.2	18.5
9	$\mu = 0.8$	-112.8	19.9
10	$\mu = 0.9$	-112.7	20.0
11	$\mu = 1$	-111.0	21.6
12	$\mu = 2$	-109.5	23.0
13	$c = 2$	-80.1	52.5
14	$c = 3$	-30.8	101.8
15	$c = 5$	40.9	173.5
16	$c = 10$	100.3	233.0
17	$c = 15$	103.4	236.0
18	$c = 20$	107.6	240.2

Second, we tested the inclusion of random effects and of different covariate structures (Table C). The first comparison was between our best model (first row in Table C) against a model with no covariates (Model 19). We then analyzed multiple combinations of different covariates (Table C) and removing random effects from different parameters. The final model, includes random effects in all parameters, except those that were fixed (μ, and c) and those which were estimated at the population level (ρ and c_A). In addition, we found support for a covariate of drug dose on drug effectiveness, and covariate of HBeAg status on β, α and δ. The best model also strongly favored a negative correlation ($r = -0.945$) between α and β, as shown by the degradation in the quality of fit when we

removed this correlation (Model 36).

Table C. Statistical models. Negative log-likelihood and corrected Bayesian information criteria (BICc) for different statistical models based on correlation, covariates and random effects. These fits were performed based on the best model (first row) with variations as described. We estimated c_A without random effects throughout the study.

**Model no.**	**Description**	**Negative log-likelihood**	**BICc**
Best model	$\mu = 0$, $c = 1$, covariate on $\varepsilon, \beta, \alpha, \delta$, random effects on all parameters except ρ, and a correlation between β and α	-120.5	12.1
19	No covariates	-42.1	73.7
20	Covariate on dose for drug effectiveness (ε) only	-51.3	71.2
21	Covariate on HBeAg status for δ only	-64.9	61.0
22	Covariate on HBeAg status for δ and α	-55.7	73.6
23	Covariate on HBeAg status for β and α	-98.9	30.4
24	No covariate on dose for drug effectiveness (ε)	-103.7	22.2
25	Covariate on HBeAg status for π	-118.7	17.3
26	Covariate on HBeAg status for π but no covariates on β or α	-64.6	64.6
27	No covariate on β	-77.4	51.9
28	No random effects on A_0	289.7	418.9
29	No random effects on A_N	-82.9	46.4
30	No random effects on β	-44.2	84.9
31	No random effects on α	-6.9	118.9
32	No random effects on δ	-103.8	28.7
33	No random effects on π	-14.0	115.3
34	Model with random effects on ρ	-115.0	21.0
35	Model with random effects on ρ but no random effects on π	-107.8	24.9
36	No correlation between β and α	-80.4	48.8

Finally, we tried several alternative model structures (Table D). In particular, we tested

i) allowing the same vs. different clearance rates for HBV RNA (c_r) and HBV DNA (c_v);

ii) allowing different export rates for encapsidated pgRNA (ρ_r) and rcDNA (ρ_v);

iii) a model (Model 43) where at the end of treatment, the drug washout was instantaneous, and so $\varepsilon = 0$ for $t \geqslant 28$ days;

iv) a model with an additional effect of treatment in reducing the secretion rates of HBV RNA and HBV DNA, i.e., changing the parameter ρ to $(1 - \varepsilon_s)\rho$ (Model 44), where $0 \leqslant \varepsilon_s \leqslant 1$ is the effectiveness of the drug in reducing the secretion of viral and HBV RNA particles.

We also analyzed two models to test the effect of incomplete reverse transcription of encapsidated pgRNA. In both we assumed that only a fraction f of the encapsidated pgRNA is successfully reverse transcribed. To implement

this, we multiplied the reverse transcription rate, π, by f in the C equation to indicate that only a fraction f of encapsidated pgRNA is reverse transcribed into rcDNA successfully. The two models differ in what we assume happens to the encapsidated pgRNA not successfully reverse transcribed into rcDNA. In the first version we assume it is destroyed, for example, by the action of RNase H which degrades pgRNA, at rate $\pi(1-f)P$ (in the P equation) and thus leads to its removal from the model, where f is the fraction of pgRNA that is successfully reverse transcribed to rcDNA. Here, we assume that RNase H activity is still present at a rate proportional to the reverse transcription rate, in spite of reverse transcription not being fully successful at generating rcDNA. Further, the successfully reverse transcribed pgRNA is also removed from the P equation and thus we leave the term $-\pi P$ in the P equation unchanged. We evaluated this scenario with (Model 45) and without (Model 46) random effects on the new parameter f. In the second version of the model with incomplete reverse transcription, we still have the term $-\pi P$ in the P equation, but added an explicit compartment to keep track of the encapsidated pgRNA that is incompletely reverse transcribed. Here, we assume this encapsidated pgRNA is then assembled into HBV RNA particles and exported. Thus, the new equation for the exported encapsidated pgRNA is $dP_E/dt = \pi(1-f)P - (\delta+\rho)P_E$. The C equation is $dC/dt = \pi f P - (\delta+\rho)C$. In this version, P is not exported, because it either is reverse transcribed into C and exported as virions, V, or incompletely reverse transcribed and tracked as P_E, which is exported as HBV RNA, R. Again, we tried two scenarios with (Model 47) and without (Model 48) random effects on the parameter f.

We next tried simple models where proliferation of both uninfected and infected cells was included (Model 49). In HBV infection, it is thought that proliferation leads to loss of cccDNA [11] and thus to infected cells becoming uninfected. This was implemented by including a term $-pI$ in the infected cell equation, and corresponding terms in the equations for P ($-pP$) and C ($-pC$), and changing the T equation by adding pT to account for proliferation of uninfected cells and adding $2pI$ to account for proliferation of infected cells leading to uninfected daughter cells. Here, we assumed that infected cell division leads to the loss of replicative intermediates (P and C) in the daughter cells, which is an approximation as some of these intracellular intermediates may be inherited.

We also studied models with non-cytolytic cure of infected cells as was done by Lewin et al. [12]. Here, we included a term $-\omega I$ in the I equation, and the corresponding term ωI in the T equation, and assumed, as an approximation, that non-cytolytic cure had a negligible effect in the overall concentration of intracellular pgRNA, P, and rcDNA, C. The idea is that non-cytolytic cure of infected cells corresponds to the loss of cccDNA in the nucleus, but this could happen without an immediate effect on cytoplasmic pgRNA and rcDNA. We further tested two versions of this non-cytolytic model, one where infected cells still died at rate δ, which is larger than the death rate of uninfected cells (Model 51) due to some cytolytic effects, and another version where there are no cytolytic effects, just infected cell "cure" (non-cytolytic), and hence infected cells are lost at the same rate as uninfected cells, replacing δ with d_T in the equations for I, P, and C (Model 53). We found a large difference between the estimated

Table D. Models with alternative mechanisms. Negative log-likelihood and corrected Bayesian information criteria (BICc) for models with alternative biological structure. These models were based on the best model (first row) with the changes stated.

Model no.	Description	Negative log-likelihood	BICc
Best model	$\mu = 0$, $c = 1$, covariate on $\varepsilon, \beta, \alpha, \delta$, random effects on all parameters except ρ, and correlation between β and α	-120.5	12.1
37	Estimate $c_r = c_v = c$ with random effects	-117.0	25.6
38	Model 37 with no random effects on c	-113.0	26.1
39	Different clearance rates for HBV RNA (c_r) and HBV DNA (c_v) with random effects	-117.3	35.2
40	Model 39 with no random effects on c_r or c_v	-118.2	27.6
41	Different export rates for encapsidated RNA (ρ_r) and rcDNA (ρ_v) with random effects	-117.0	28.9
42	Model 41 with no random effects on ρ_r and ρ_v	-119.0	20.3
43	Model with drug effectiveness $\varepsilon = 0$ after treatment interruption	-114.4	8.3
44	Model with drug effect on virus secretion rate (ρ)	-111.7	37.7
45	Model with incomplete reverse transcription (version 1). Estimate fraction of pgRNA that complete reverse transcription to rcDNA (f) with random effects	-119.2	23.3
46	Model 45 with no random effects on f	-116.5	22.7
47	Model with incomplete reverse transcription (version 2). Estimate f with random effects	-113.1	29.5
48	Model 47 with no random effects on f	-9.0	130.2
49	Model with cell-division. Estimate cell-division rate p with random effects	-116.1	19.9
50	Model 49 with no random effects on cell-division rate (p)	-2.1	130.5
51	Model with non-cytolytic cure (version 1). Estimate cure rate (ω) with random effects	-118.8	23.8
52	Model 51 with no random effects on ω	-111.2	28.0
53	Model with non-cytolytic cure (version 2), with same death rate for infected and uninfected cells . Estimate ω with random effects	383.7	516.3
54	Model 53 with no random effects on ω	385.9	515.2
55	Estimate production rate of uninfected hepatocytes, λ, with a covariate for HBeAg and no covariate on β	-55.1	89.0
56	Model 55 with no random effects on λ	-53.5	87.3
57	Model with no ALT dynamics	–	–

β for HBeAg-positive and HBeAg-negative infected individuals, and wondered if our assumption that the source rate of target cells, λ is fixed and equal between the two types of infection could be responsible. Thus, we tested a model with the same β for HBeAg-positive and HBeAg-negative (*i.e.* no covariate for this parameter) and estimating λ with a covariate (Models 55 and 56 in Table D). In these fits, we keep $d_T = 0.004$/day as in the main model, but now fit λ with a HBeAg-status dependent covariate, and re-calculate T_0 as λ/d_T. In this model, T would be interpreted not as all uninfected hepatocytes, but only a subset, presumably those that can be infected (*e.g.* are not protected by an immune response).

Finally, we tested a model where we did not fit ALT, just HBV RNA and HBV DNA (Model 57 in Table D). As the data being fit is different, this model can't be compared in terms of BICc with the others.

The results of fitting all these extra models are shown in Table D. We found that most of these models did not provide an improved fit, as measured by BICc, over the model presented in detail in the main text ("Best model" in Table D). The exception was the model with instantaneous drug washout ($i.e.$, $\varepsilon = 0$ following treatment cessation, Model 43), which we did not adopt because it is not biologically realistic.

Overall, there were, however, a few models that still provided reasonable fits to the data (as measured by BICc). As a further test of the robustness of our results, we present in Figs F and G a comparison of the estimated population parameters for the top nine models plus the model without ALT (Model 57), which demonstrates that the population parameter estimates are very similar across these models.

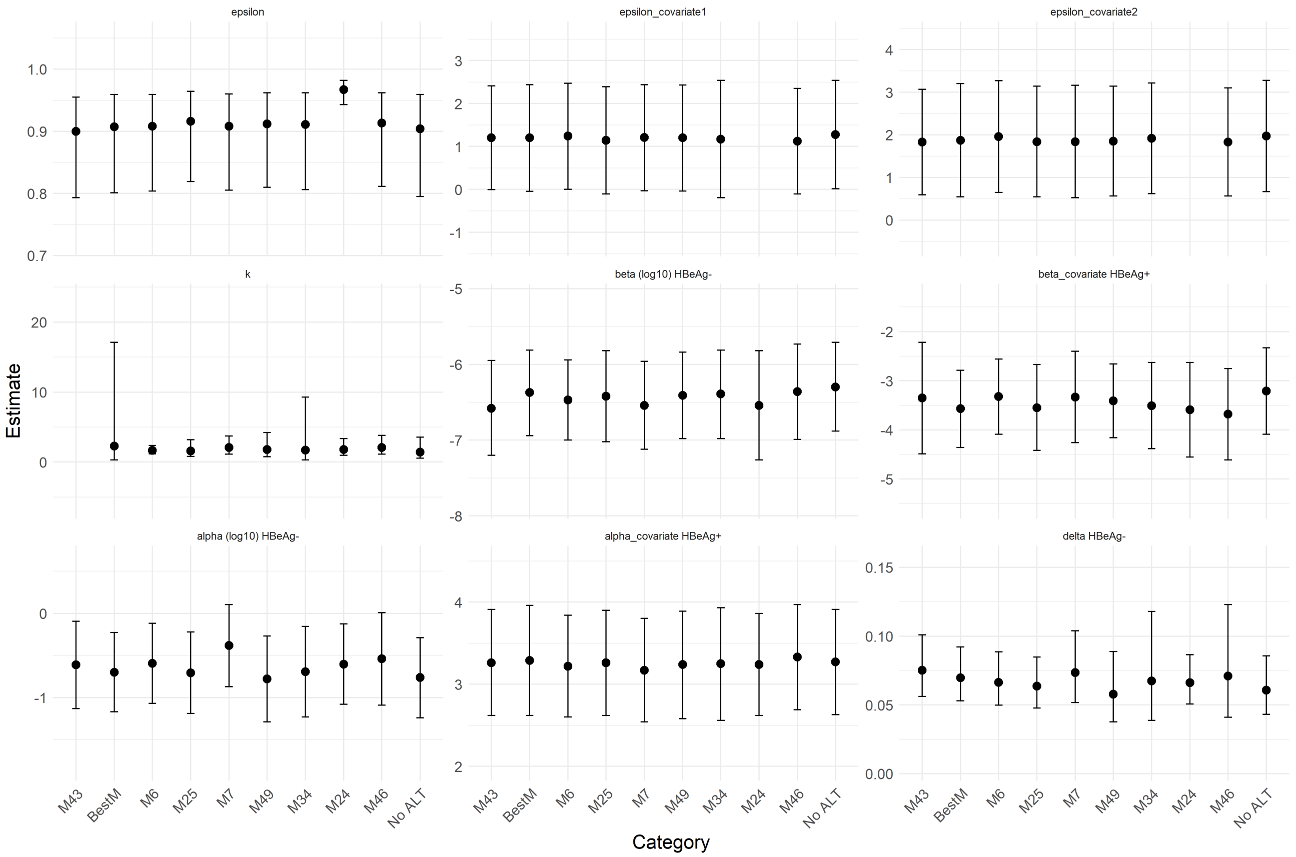

Fig F. Comparison of the population estimates for the different model parameters (panels) for the top nine models (x-axis). The model numbers are shown in Tables B, C, and D, and the models are ordered from smallest BICc to highest (plus the no ALT model). The population parameter estimates, shown as dots with the 95% confidence interval in these estimates as calculated by Monolix, are very similar across all these models.

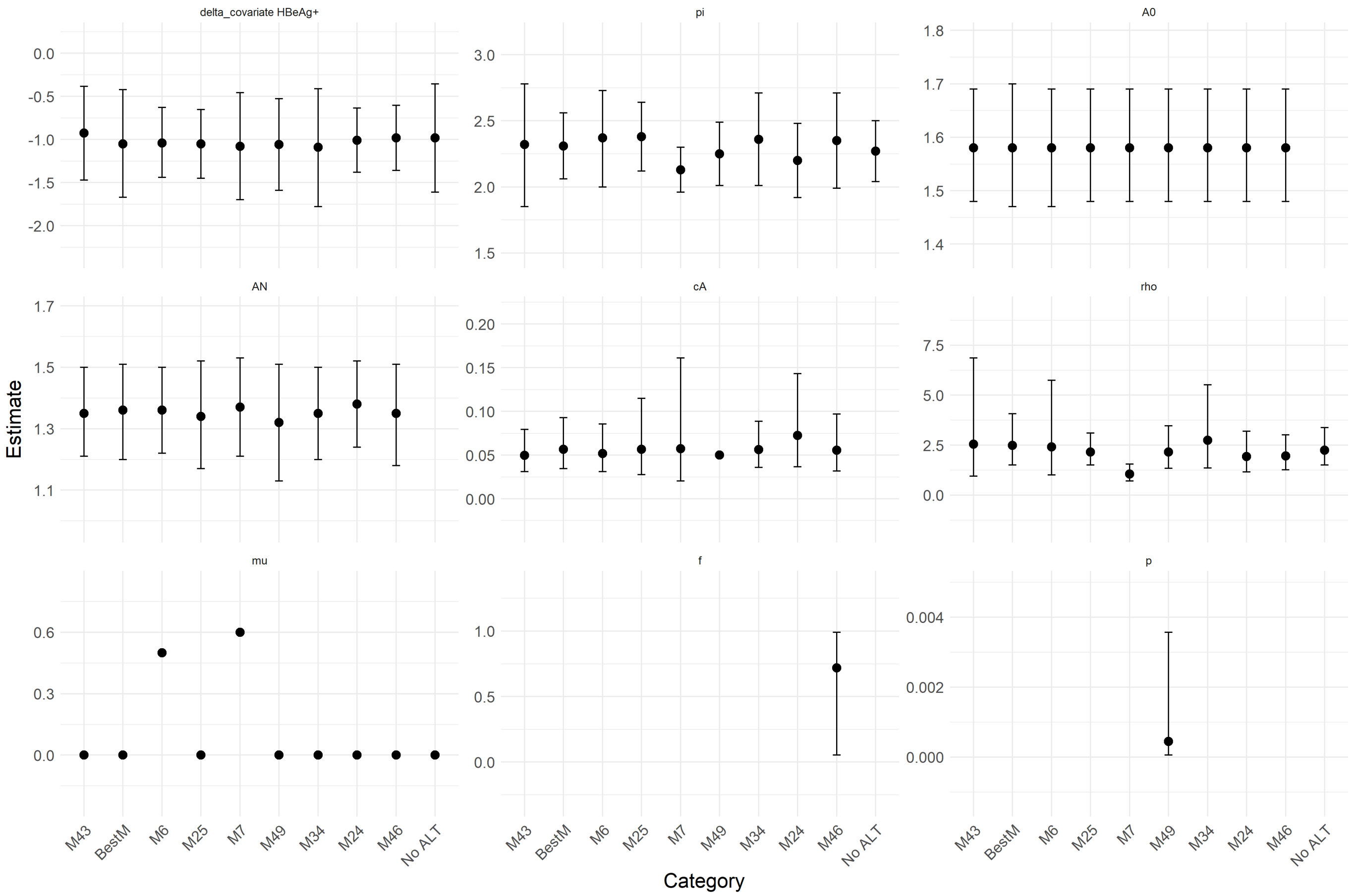

Fig G. Comparison of the population estimates for the different model parameters (panels) for the top nine models (x-axis), continuation. The model numbers are shown in Tables B, C, and D, and the models are ordered from smallest BICc to highest (plus the no ALT model). The population parameter estimatesshown as dots with the 95% confidence interval in these estimates as calculated by Monolix are very similar across all these models. Note that μ is always fixed.

Parameter identifiability analysis

We now show that the HBV RNA and HBV DNA dynamics are sufficient to identify the unknown model parameters for the best model in Table B. As we have shown, the observable dynamics of HBV RNA and HBV DNA during treatment are primarily driven by a biphasic decline with rates corresponding to the natural clearance rate c and the death rate of infected hepatocytes, δ. Consequently, these two parameters are well informed by the dynamics of circulating HBV RNA, HBV DNA, and ALT. As stated in the main text, both λ and d_T were fixed based on published literature about the number of hepatocytes in the liver and their lifespan (except in the case of our alternative Models 55 and 56 above). We now show that the remaining model parameters are informed by available viral load data.

First, we recall that we imposed that the initial conditions of Eq. (S2) correspond to the infected equilibrium in

Eq. (S6) before treatment was initiated. We fixed $c_v = c_r = 1$/day and $\mu_v = \mu_r = 0$. Next, if we assume that the reverse transcription rate, π, is significantly larger than the rates of secretion ρ, degradation, μ, and death of infected cells, δ, then $\psi_1 = \pi + \rho + \mu + \delta \approx \pi$. This assumption is satisfied in all of our parameter estimations (cf. Table A) and in a similar multiscale model of chronic HBV [5].

With these approximations and the assumption $\mu = 0$, using the expressions for $R(0)$ and $V(0)$ given in Eq. (S6), we find

$$\frac{V(0)}{R(0)} = \frac{\pi}{\rho + \delta}.$$

This ratio is similar to prior work [5], where the ratio of baseline HBV DNA to HBV RNA was reported as approximately the rate of reverse transcription, π. There was no significant difference in the ratio of $V(0)/R(0)$ between HBeAg-positive and HBeAg-negative individuals. Thus, this analysis indicates that the rate of reverse transcription, π, is not HBeAg-dependent. To further investigate the potential impact of HBeAg status on the rate of reverse transcription, we also tested alternative models (Models 25 and 26) with a covariate on π. These models did not provide an improved fit the data compared against the best model

We now analyze in more detail the initial decay of HBV DNA and HBV RNA. The observable initial decay of circulating HBV RNA is entirely driven by the clearance rate c. However, the HBV DNA dynamics are not purely exponential during the first few days of treatment. Rather, there is a slight lag between treatment initiation at time $t = 0$ and the beginning of exponential clearance with rate c (see Fig H). This lag is due to the presence of intracellular rcDNA that was formed prior to treatment initiation that can be packaged into viral particles and secreted from infected hepatocytes even after treatment initation. However, following treatment initiation, this intracellular rcDNA decays with rate $\psi_2 = \delta + \rho + \mu$, which is approximately 2.5/day (cf. Table A) and thus this effect is short-lived. This secretion of rcDNA as viral particles accounts for the lag between treatment initiation and the beginning of HBV DNA exponential decay with rate c. Conversely, intracellular encapisdated pgRNA reaches the treated quasi steady-state almost immediately (at rate $\psi_1 \approx 200$/day) upon treatment initiation, so there is no lag for HBV RNA, which then decays at the clearance rate c=1/day. Consequently, the difference in relative HBV RNA and HBV DNA dynamics during the first few days of treatment identifies $\psi_2 = \rho + \delta + \mu$. As $\mu = 0$ and δ is determined by the final phase of decline of both HBV RNA and HBV DNA, we can thus estimate ρ. Indeed, the mean relative change in the observed HBV RNA data between baseline and day 7 for participants in the vebicorvir trial with both HBV RNA and HBV DNA measurements is -1.33 $\log_{10}$ while the corresponding mean change in the observed HBV DNA concentration is -1.18 $\log_{10}$.

Next, we show how the baseline HBV RNA concentration directly informs our estimates for α. We recall that we

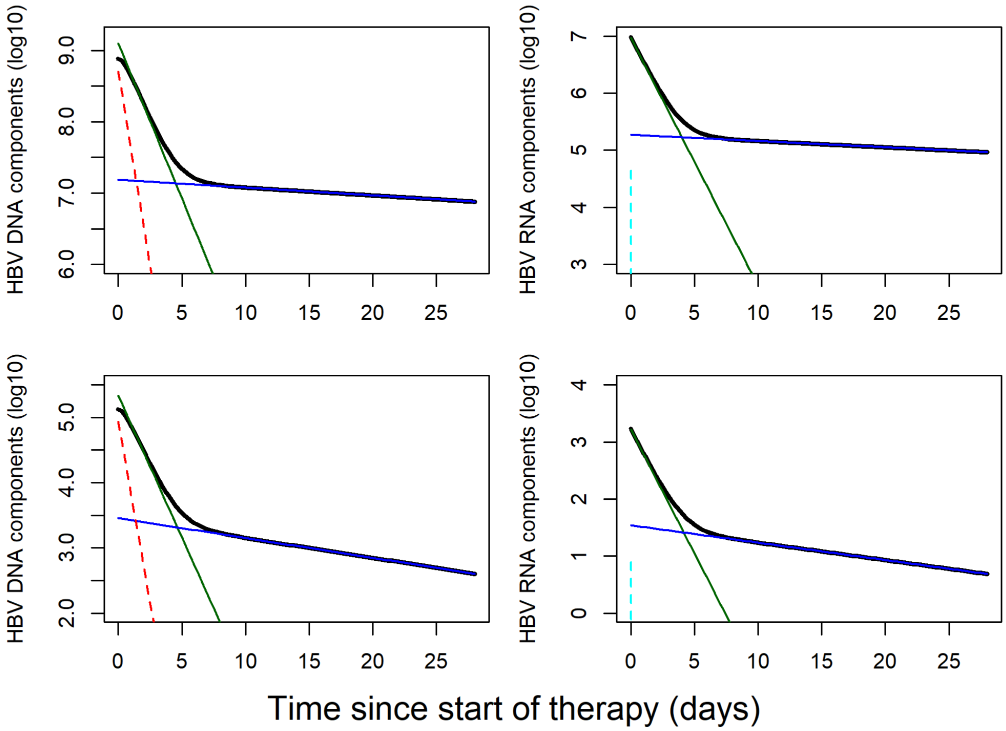

Fig H. Comparison of decay of the different components in HBV DNA (left) and HBV RNA (right) for HBeAg-positive infection (top) and HBeAg-negative infection (bottom) . Each line corresponds to an exponential component of the decay of these variables according to the approximation in Eqs. (S18) and (S17). The black thick line is the decay of total HBV DNA (left) or HBV RNA (right), the green line is the term in e^{-ct}, the blue line is the term in $e^{-\delta t}$, the cyan line, which is close to the y-axis, is the term in $e^{-\psi_1 t}$ (only seen in the HBV RNA plots, as it is too small in the HBV DNA plots), and the red line the term in $e^{-\psi_2 t}$, which does not exist in the HBV RNA decay. Note that dashed lines correspond to negative values, so for example the viral load (HBV DNA, black line) is the sum of the green line plus the blue line minus the red dashed line. The term in $e^{-\psi_1 t}$ has a negligible contribution, because it decays at rate $\psi_1 > \pi \approx 200$/day.

fixed λ, and that our prior analysis links ρ, δ, and π to observable viral dynamics. The baseline HBV RNA concentration is given by

$$R(0) = \frac{\lambda \rho \alpha}{c \delta \pi}.$$

As all parameters defining $R(0)$ except α, and λ which is fixed, are determined by the dynamics of HBV RNA and HBV DNA during treatment, the baseline HBV RNA concentration informs estimates of α. Furthermore, as δ can be estimated from the final phase of HBV DNA decay, the ratio of $R(0)$ between HBeAg-positive and HBeAg-negative infection informs the difference of α between these two forms of infection. We identified a significant difference between the baseline HBV RNA concentration for HBeAg-positive and HBeAg-negative participants ($p = 5 \times 10^{-5}$

using the Mann-Whitney test) with $R(0)$ found to be about 2000 times larger for HBeAg-positive infection.

Finally, we consider the rate of viral rebound during the first week following treatment cessation. We assume that the uninfected hepatocytes remain approximately constant during this period, so $T(t) = T_\tau$, for $t \in (\tau, \tau + 7)$ where τ is the time of treatment cessation at 28 days. Then, assuming that the concentration of infected hepatocytes is in quasi-steady state with circulating HBV DNA during the first week following treatment interruption, we find

$$I_\tau(t) = \frac{\beta V(t) T_\tau}{\delta}.$$

Next, we assume that the intracellular dynamics are significantly faster than the extracellular dynamics and that the CAM effect washes out quickly following treatment cessation, so imposing quasi-steady state for P and C gives

$$P_\tau(t) = \frac{\alpha \beta T_\tau}{\delta \psi_2} V(t) \quad \text{and} \quad C(t) = \frac{\pi \alpha \beta T_\tau}{\delta \psi_2 \psi_1} V(t).$$

Recalling that

$$\frac{\mathrm{d}}{\mathrm{dt}} V(t) = \rho_v \, C(t) - c_v \, V(t),$$

the HBV DNA dynamics satisfy

$$\frac{\mathrm{d}}{\mathrm{dt}} V(t) = c \left[\left(\frac{\rho}{\rho + \delta} \right) \left(\frac{\alpha \beta T_\tau}{\delta c} \right) - 1 \right] V(t).$$

The level of uninfected hepatocytes, T_τ, is a dynamical variable tracked by the model and all other parameters can be identified as described above, so the rate of viral rebound following treatment interruption, which is observable in our viral load data, informs the infection rate β. The rate of viral rebound is directly related to the basic reproduction number, $\mathcal{R}_0$, given in Eq. (S9) under the assumption that T_τ is the hepatocyte concentration corresponding to the uninfected equilibrium. Specifically, recalling that $\psi_1 \approx \pi$ and setting $T_\tau = \lambda / d_T$, then the rate of viral rebound is $c(\mathcal{R}_0 - 1)$.

The population estimates for α differ over three orders of magnitude and are directly informed by the baseline HBV RNA concentration, with HBeAg-positive participants having a larger baseline HBV RNA concentration and corresponding production rate of encapsidated pgRNA. To reflect the relatively small differences in the speed of viral rebound, the population estimates for β must also differ over about 3 orders of magnitude, with HBeAg-positive participants having a lower infection rate, β.

We also used the likelihood continuation technique from [23] to quantify how sensitive the estimated parameters

are to the sampled data. This approach corresponds to perturbing the experimental data by a small amount, then predicting the maximum likelihood estimate for this perturbed data. Consequently, we can use this continuation technique to quantify how each individual's parameter estimates depend on the viral load data. In Fig I, we show the median relative change in the maximum likelihood estimator for each parameter predicted as the result of a 10% perturbation in each HBV RNA and HBV DNA data point. We perturbed each of the 5 HBV RNA and HBV DNA measurements during treatment, at days 1, 7, 14, 21, and 28, and predicted how each of the fit parameters would change in response to this perturbation in the data. We note that changes in both HBV RNA and HBV DNA measurements on days 14, 21, and 28 strongly influence the estimate of ε. These measurements occur during the second phase of HBV RNA and HBV DNA decline. The primary effect of treatment, and thus ε, drives the duration of the first phase of decline: increases in ε result in a longer first-phase of decline with a corresponding increase in the relative decay of HBV RNA and HBV DNA during the first phase of decline. Consequently, perturbations in HBV RNA and HBV DNA measurements on days 14, 21, and 28 are predicted to modulate the duration and depth of first phase of decline via changes in ε. We also note that ε is more sensitive to perturbations in HBV RNA than HBV DNA data, which further supports our conclusion that HBV RNA is an informative biomarker of CAM effectiveness, as indicated by the relative influence of perturbations in the HBV RNA data compared against HBV DNA.

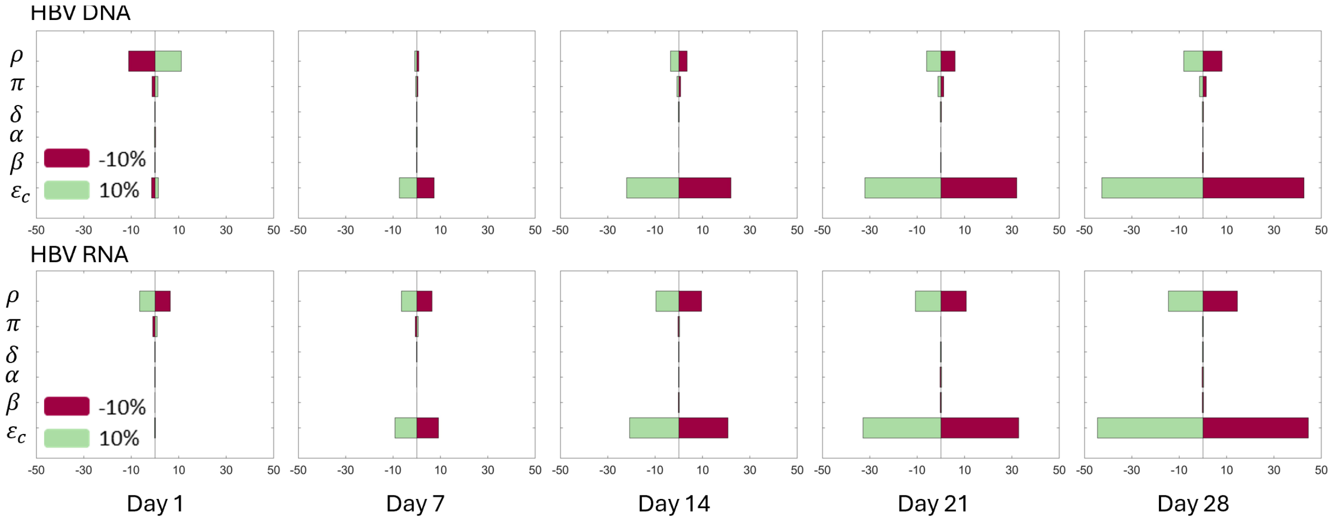

Fig I. Sensitivity of the maximum likelihood estimator to perturbations in viral data. (A) The top row shows the median predicted change in the model parameters if the sampled HBV DNA concentration is perturbed by ±10% from the true value. The bottom row shows the median predicted change in the model parameters if the sampled HBV RNA concentration is perturbed by ±10% from the true value. The various subpanels represent samples taken on days $1, 7, 15, 21$, and 28 post-treatment initiation.

Supplementary Figures for the Main Text

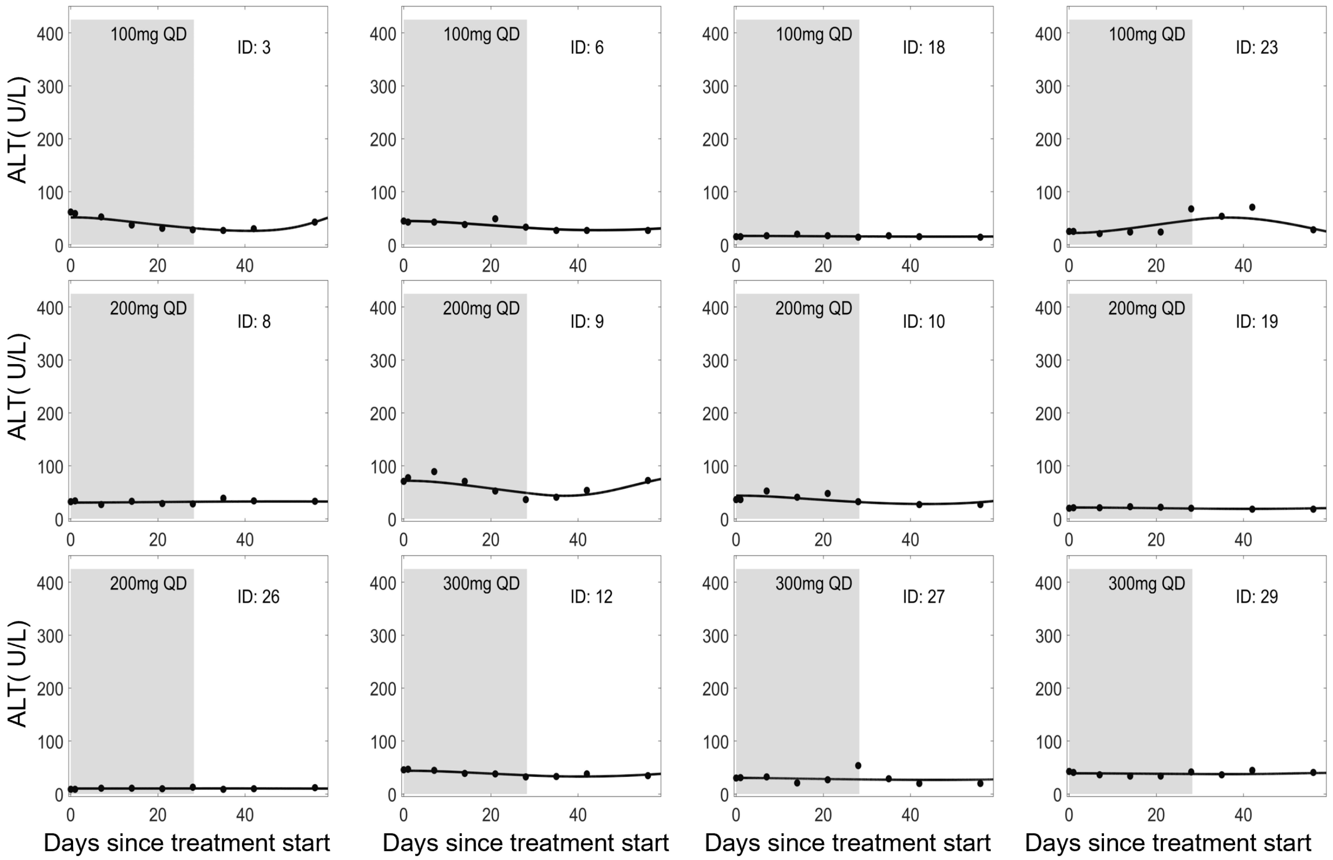

Fig J. Model fits of ALT during treatment period and follow-up (HBeAg-negative group). Corresponding HBV DNA and HBV RNA fits for these participants are given in Fig 3. The shaded region represents the treatment period while the unshaded region is for follow-up. Dots are viral measurements and solid lines are model predictions.

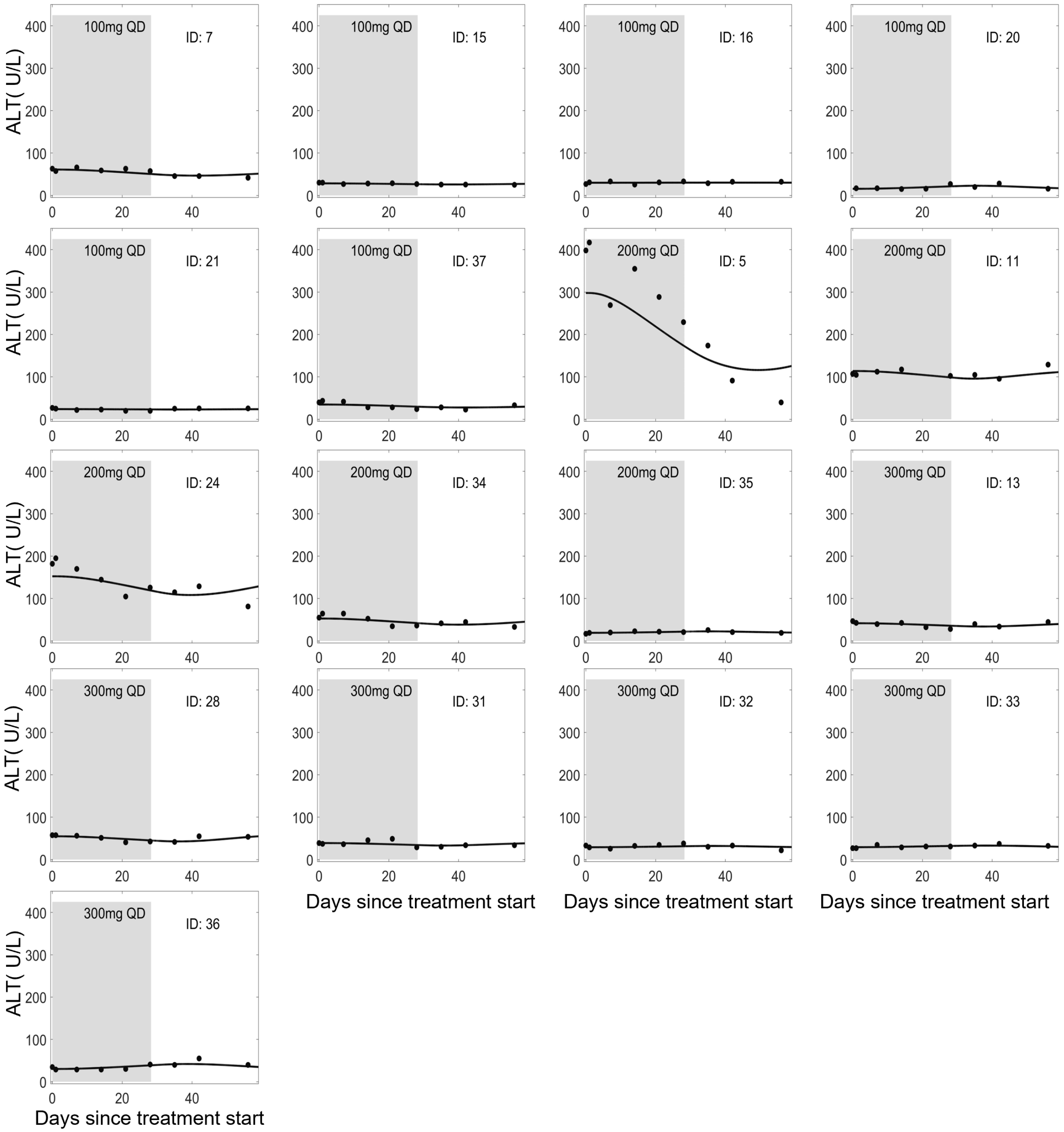

Fig K. Model fits of ALT during treatment and follow-up (HBeAg-positive group). Corresponding ALT fits for the results in Fig 4. The shaded region represents the treatment period while the unshaded region is for follow-up. Dots are viral measurements and solid lines are model predictions.

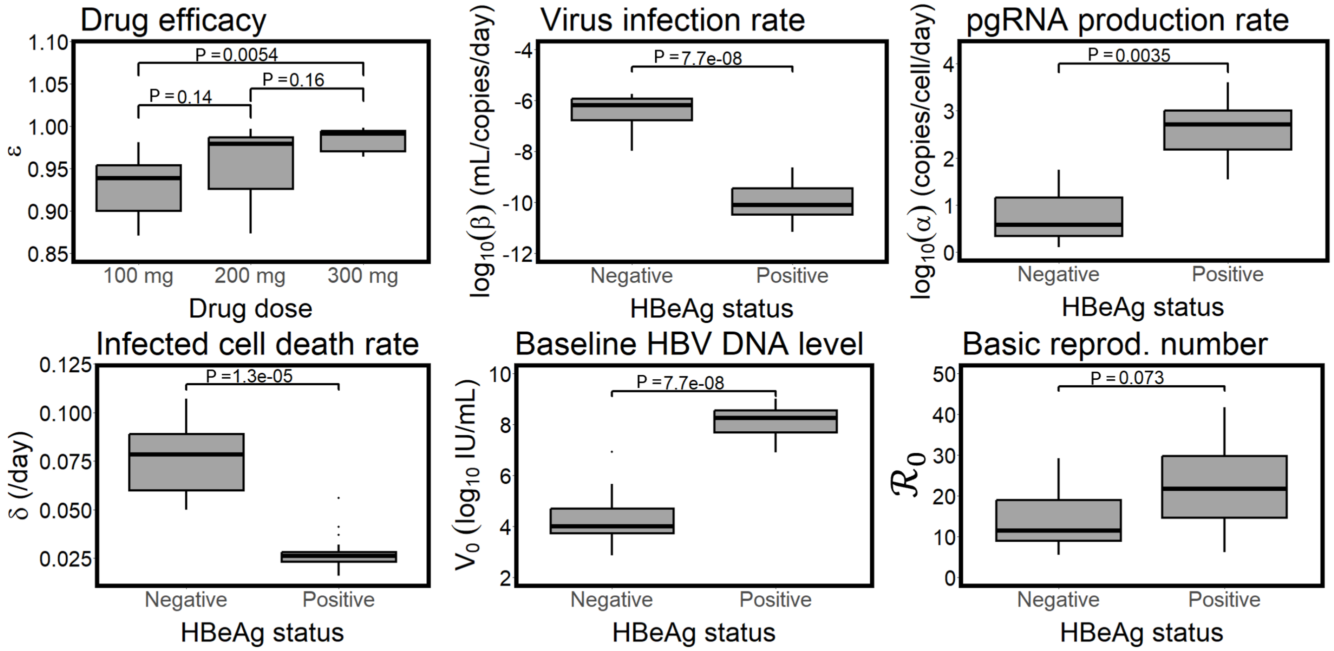

Fig L. Distribution of estimated individual parameters. Boxplots of estimated drug efficacy for different doses of vebicorvir (top left), and estimated virus infectious rate, β (mL/copies/day, top middle), encapsidated pgRNA production rate, α (copies/cell/mL/day, top right), infected cell death rate, δ (/day, bottom left), baseline HBV DNA level, V_0 (IU/mL, bottom middle) and basic reproduction number, $\mathcal{R}_0$ (bottom right) (in $\log_{10}$) by HBeAg status. Each plot shows the distribution of the estimated individual parameters from the model fit to the complete data (Figs 3, 4, J and K). P-values (shown on the brackets) were computed using the Wilcoxon test [19] package in R version 3.6.3 [20], and compare the means of the respective distributions.

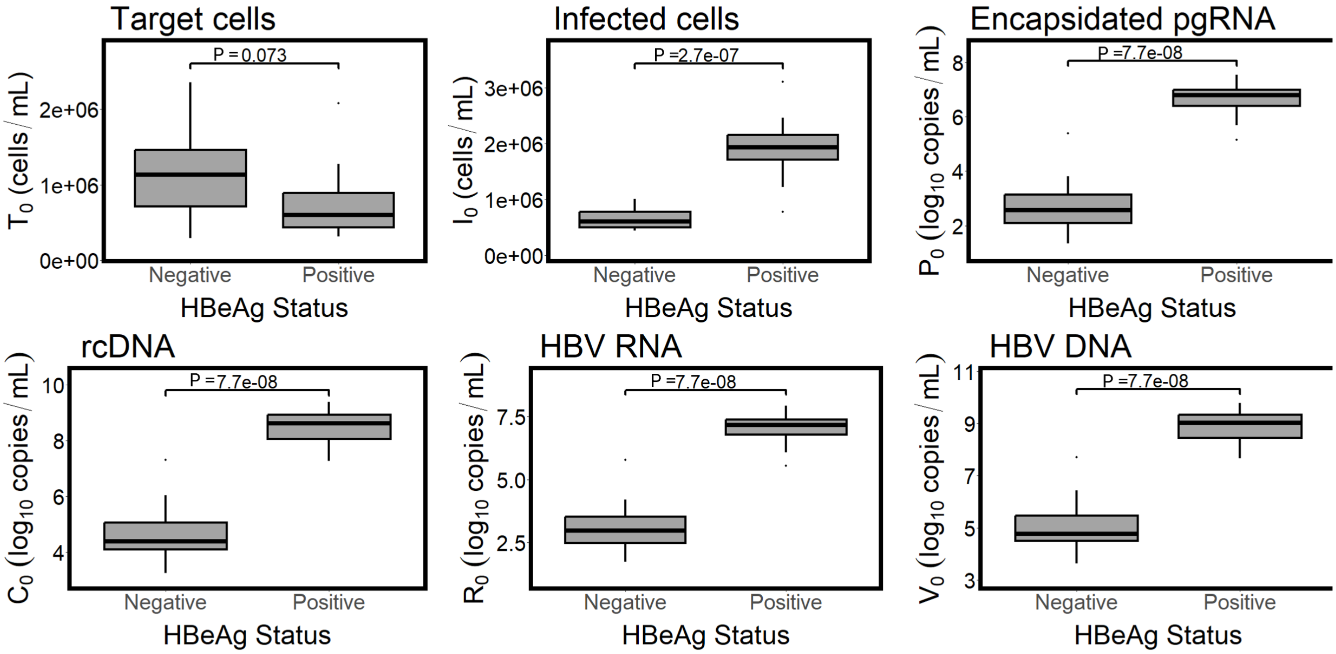

Fig M. Predicted pre-treatment steady-states. Boxplots of predicted pre-treatment steady-state concentration of uninfected hepatocytes (top left), infected hepatocytes (top middle), intracellular encapsidated pgRNA (top right), intracellular rcDNA (bottom left), HBV RNA (bottom middle) and HBV DNA (bottom right). The steady-state distribution for each quantity was computed with Eq. (S5) using the estimated individual parameters from the model fits. There was no significant difference in the pre-treatment ALT steady-state. P-values (shown on the brackets) were computed using the Wilcoxon test [19] package in R version 3.6.3 [20] and comparing the means of the respective distributions.

Monolix model used for data fitting

```
[LONGITUDINAL]

input = {e_c, k, beta_exp, alpha_exp,  delta, pi_exp,  A0,  AN, cA, rho_v, rho_r,
c_v, c_r,  mu_v, mu_r};


EQUATION:

odeType = stiff


; implementing treatment

if t < 28 ; during therap

  eps_cpam = e_c;

else ; post therapy

z = exp(-k*(t - 28))

eps_cpam = (e_c*z)/( e_c*(z - 1) + 1) ;

end


t0 = 0

dT = 0.004;

lambda = dT*13000000 ;

s = 10^(AN)*cA;

alpha = 10^alpha_exp

pi = 10^pi_exp

beta = 10^beta_exp


M = (pi*alpha)/(delta*(mu_v + delta + rho_v)*(mu_r + delta + pi + rho_r) );

T_e = c_v/(rho_v*beta*M);

I_e = lambda/delta - (c_v*dT)/(beta*delta*rho_v*M);

P_e = (alpha/(mu_r + delta + pi + rho_r) )*I_e;

C_e = lambda*M - (c_v*dT)/(beta*rho_v);

R_e = ( (alpha*rho_r)/( c_r*(mu_r + delta + pi + rho_r) ))*I_e;

V_e = lambda*rho_v*M/c_v  - dT/beta;
```

```
V_0 = V_e;
T_0 = T_e;
I_0 = I_e;
P_0 = P_e;
C_0 = C_e;
R_0 = R_e;
A_0 = 10^(A0);
alphaA = (cA*10^(A0) - s)/(delta*I_0);

ddt_T = lambda - dT*T - beta*T*V
ddt_I  = beta*T*V - delta*I
ddt_P = (1 - eps_cpam)*alpha*I -  (mu_r + rho_r + (1 - eps_na)*pi + delta)*P ;
ddt_C =  (1 - eps_na)*pi*P -  (mu_v + rho_v + delta)*C;
ddt_R = rho_r*P - c_r*R;
ddt_V = rho_v*C - c_v*V;
ddt_A = s + alphaA*delta*I - cA*A;

ValueDNA = log10( max(0.0000001, V/5.82) )
ValueRNA = log10( max(0.0000001, R) )
ValueALT = log10( max(0.0000001, A) )

OUTPUT:
output = {ValueDNA,ValueRNA,ValueALT}
```

References

1. van den Driessche P, Watmough J. Reproduction numbers and sub-threshold endemic equilibria for compartmental models of disease transmission. Math Biosci. 2002;180:29–48. doi:10.1016/S0025-5564(02)00108-6.

2. Diekmann O, Heesterbeek JAP, Metz JAJ. On the definition and the computation of the basic reproduction ratio R 0 in models for infectious diseases in heterogeneous populations. J Math Biol. 1990;28:365–382. doi:10.1007/BF00178324.

3. Rong L, Perelson AS. Mathematical analysis of multiscale models for hepatitis C virus dynamics under therapy with direct-acting antiviral agents. Math Biosci. 2013;245:22–30. doi:10.1016/j.mbs.2013.04.012.

4. Cardozo EF, Ji D, Lau G, Schinazi RF, Chen G, Ribeiro RM, et al. Disentangling the lifespans of hepatitis C virus–infected cells and intracellular vRNA replication–complexes during direct-acting anti–viral therapy. J Viral Hepat. 2020;27:261–269. doi:10.1111/jvh.13229.

5. Gonçalves A, Lemenuel-Diot A, Cosson V, Jin Y, Feng S, Bo Q, et al. What drives the dynamics of HBV RNA during treatment? J Viral Hepat. 2021;28:383–392. doi:10.1111/jvh.13425.

6. Guedj J, Dahari H, Rong L, Sansone ND, Nettles RE, Cotler SJ, et al. Modeling shows that the NS5A inhibitor daclatasvir has two modes of action and yields a shorter estimate of the hepatitis C virus half-life. Proc Natl Acad Sci. U.S.A. 2013;110:3991–3996. doi:10.1073/pnas.1203110110.

7. Kitagawa K, Nakaoka S, Asai Y, Watashi K, Iwami S. A PDE multiscale model of hepatitis C virus infection can be transformed to a system of ODEs. J Theor Biol. 2018;448:80–85. doi:10.1016/j.jtbi.2018.04.006.

8. Kitagawa K, Kuniya T, Nakaoka S, Asai Y, Watashi K, Iwami S. Mathematical Analysis of a Transformed ODE from a PDE Multiscale Model of Hepatitis C Virus Infection. Bull Math Biol. 2019;81:1427–1441. doi:10.1007/s11538-018-00564-y.

9. Kitagawa K, Kim KS, Iwamoto M, Hayashi S, Park H, Nishiyama T, et al. Multiscale modeling of HBV infection integrating intra- and intercellular viral propagation to analyze extracellular viral markers. PLoS Comput Biol. 2024;20:e1011238. doi:10.1371/journal.pcbi.1011238.

10. Burnham KP, Anderson DR. Practical Use of the Information-Theoretic Approach. In: Model Selection and Inference. vol. 61. New York, NY: Springer New York; 1998. p. 75–117. Available from: `http://link.springer.com/10.1007/978-1-4757-2917-7{_}3`.

11. Thomas T, Benno Z, Jochen W, Henrik Z, Sally C, Vikki H, et al. Mitosis of hepatitis B virus-infected cells in vitro results in uninfected daughter cells. JHEP Reports 4:100514. doi:10.1016/j.jhepr.2022.100514.

12. Lewin, S. R., Ribeiro, R. M., Walters, T., Lau, G. K., Bowden, S., Locarnini, S., and Perelson, A. S. (2001). Analysis of hepatitis B viral load decline under potent therapy: Complex decay profiles observed. *Hepatology*. 2001; 34:1012–1020.

13. Monolix 2021R1, Lixoft SAS, a Simulations Plus company

14. Lavielle M. Mixed Effects Models for the Population Approach. Chapman and Hall/CRC; 2014. Available from: `https://www.taylorfrancis.com/books/9781482226515`.

15. Zitzmann C, Ke R, Ribeiro RM, Perelson AS. How robust are estimates of key parameters in standard viral dynamic models? PLoS Comput Biol. 2024;20:e1011437. doi:10.1371/journal.pcbi.1011437.

16. Cao Y, Gao W, Caro L, Stone JA. Immune-viral dynamics modeling for SARS-CoV-2 drug development. Clin Transl Sci. 2021;14:2348–2359. doi:10.1111/cts.13099.

17. Clairon Q, Prague M, Planas D, Bruel T, Hocqueloux L, Prazuck T, et al. Modeling the kinetics of the neutralizing antibody response against SARS-CoV-2 variants after several administrations of Bnt162b2. PLoS Comput Biol. 2023;19:e1011282. doi:10.1371/journal.pcbi.1011282.

18. Marc A, Marlin R, Donati F, Prague M, Kerioui M, Hérate C, et al. Impact of variants of concern on SARS-CoV-2 viral dynamics in non-human primates. PLoS Comput Biol. 2023;19:e1010721. doi:10.1371/journal.pcbi.1010721.

19. Bauer DF. Constructing Confidence Sets Using Rank Statistics. J Am Stat Assoc. 1972;67:687–690. doi:10.1080/01621459.1972.10481279.

20. R Core Team. R: A Language and Environment for Statistical Computing; 2021. Available from: `https://www.r-project.org/`.

21. Berke JM, Tan Y, Sauviller S, Wu Dt, Zhang K, Conceição-Neto N, et al. Class A capsid assembly modulator apoptotic elimination of hepatocytes with high HBV core antigen level in vivo is dependent on de novo core protein translation. J Virol. 2024;98. doi:10.1128/jvi.01502-23.

22. Kum DB, Vanrusselt H, Acosta Sanchez A, Taverniti V, Verrier ER, Baumert TF, et al. Class A capsid assembly modulator RG7907 clears HBV-infected hepatocytes through core-dependent hepatocyte death and proliferation. Hepatology. 2023;78:1252–1265. doi:10.1097/HEP.0000000000000428.

23. Cassidy T. A continuation technique for maximum likelihood estimators in biological models. Bull Math Biol. 2023;85:90. doi:10.1007/s11538-023-01200-0.
